# Ku70 Serine 155 mediates Aurora B inhibition and activation of the DNA damage response

**DOI:** 10.1038/srep37194

**Published:** 2016-11-16

**Authors:** Victoria L. Fell, Elizabeth A. Walden, Sarah M. Hoffer, Stephanie R. Rogers, Amelia S. Aitken, Louisa M. Salemi, Caroline Schild-Poulter

**Affiliations:** 1 Robarts Research Institute and Department of Biochemistry, Schulich School of Medicine & Dentistry, The University of Western Ontario, London, Ontario N6A 5B7, Canada

## Abstract

The Ku heterodimer (Ku70/Ku80) is the central DNA binding component of the classical non-homologous end joining (NHEJ) pathway that repairs DNA double-stranded breaks (DSBs), serving as the scaffold for the formation of the NHEJ complex. Here we show that Ku70 is phosphorylated on Serine 155 in response to DNA damage. Expression of Ku70 bearing a S155 phosphomimetic substitution (Ku70 S155D) in Ku70-deficient mouse embryonic fibroblasts (MEFs) triggered cell cycle arrest at multiple checkpoints and altered expression of several cell cycle regulators in absence of DNA damage. Cells expressing Ku70 S155D exhibited a constitutive DNA damage response, including ATM activation, H2AX phosphorylation and 53BP1 foci formation. Ku70 S155D was found to interact with Aurora B and to have an inhibitory effect on Aurora B kinase activity. Lastly, we demonstrate that Ku and Aurora B interact following ionizing radiation treatment and that Aurora B inhibition in response to DNA damage is dependent upon Ku70 S155 phosphorylation. This uncovers a new pathway where Ku may relay signaling to Aurora B to enforce cell cycle arrest in response to DNA damage.

Double-strand breaks (DSBs) are the most dangerous form of DNA damage, as improperly repaired, they can result in genetic alterations, leading to genomic instability, a hallmark of cancer. Eukaryotic cells employ DNA damage checkpoint surveillance mechanisms to allow the damaged cell time to repair its DNA, or eliminate cells damaged beyond repair through apoptosis and senescence^1^,^2^.

The DNA damage response (DDR) pathway is initiated by sensor proteins that accumulate in foci at the site of damage^1^,^3^. This accumulation of DDR proteins activates a phosphorylation cascade as well as modifies surrounding chromatin to allow access of the DNA repair factors. The initial sensors include the Mre11-Rad50-NBS1 (MRN) complex, 53BP1, as well as the serine/threonine (S/T) phosphoinositide-3-kinase (PI3K) family members ATM (Ataxia Telangiectasia Mutated), and ATR (Ataxia Telangiectasia and Rad3-related). The PI3K-like kinases are the main regulators of the DDR and orchestrate many phosphorylation events at the site of DNA damage that promote DNA repair^3^. The consequences of DNA damage involve temporary cell cycle arrest to allow for DNA repair and activation of senescence or apoptotic pathways if repair cannot be completed^2^,^4^.

Aurora kinases (Aurora-A, -B and -C) are a family of serine/threonine kinases that play essential roles in cell cycle progression^5^. Aurora B functions to regulate many aspects of mitosis including chromosome-microtubule interactions, spindle assembly, sister chromatid and centromeric cohesion and cytokinesis^5^. Aurora B’s activities, however, are not restricted to mitosis, as it is expressed throughout the cell cycle and there is evidence that it also contributes to G1/S and G2/M checkpoint regulation^6^,^7^. Ectopic expression of both Aurora A and B results in chromosomal abnormalities and cellular transformation, and overexpression of Aurora kinases is observed in a number of different cancers^5^,^8^,^9^,^10^. Consequently Aurora kinases were identified as possible druggable targets and a number of Aurora kinases inhibitors have been developed for anti-cancer therapy^8^,^11^. Aurora B inhibitors were shown to prevent cytokinesis and cause cell growth inhibition and cell cycle arrest^12^,^13^,^14^. While Aurora B has been implicated in the cellular response to DNA damaging agents^15^,^16^, a thorough understanding of the regulation of Aurora kinase activity following genotoxic stress is lacking.

DSBs are repaired through three main pathways: homologous recombination (HR), which occurs primarily in the late S and G2 phases, micro-homology mediated end joining (MMEJ), a backup pathway, and non-homologous end joining (NHEJ), the predominant repair pathway in higher eukaryotes, which occurs mainly in the G1 phase^17^,^18^. The Ku heterodimer, composed of subunits Ku70 and Ku80 (70 and 86 kDa, respectively) is the initial DNA end-binding component of the NHEJ complex^19^. Each Ku subunit contains an N-terminal α/ß von Willebrand A (vWA) like domain, a central ß-barrel domain and a divergent C-terminal helical region^20^. The Ku dimer forms an asymmetrical ring lined with positively charged and hydrophobic residues that can accommodate the double-stranded DNA backbone independent of sequence^21^. Following the introduction of a DSB, Ku rapidly binds the broken ends and forms a complex with the PI3K-like kinase DNA-PKcs to recruit other NHEJ repair factors and promote repair of the break^22^. Ku also has important functions in telomere maintenance and protection, and Ku-deficiency leads to telomere defects^23^,^24^.

There is increasing evidence that the Ku N-terminal regions play important roles in NHEJ as well as in telomere maintenance and apoptotic signaling^19^. The Ku70 and Ku80 N-terminal domains have structural similarities with the von Willebrand A (vWA) domain, an ancient, evolutionarily conserved domain that is found in several extracellular and intracellular proteins, where it mediates protein-protein interactions^25^. Indeed Ku forms numerous protein interactions during NHEJ and other processes, and some of these map to the vWA domain^19^.

Our previous investigation focused on identifying key residues in the Ku70 vWA domain involved in the cellular response to DNA damage^26^. We demonstrated that mutation of Ku70 serine 155 to alanine in mouse embryonic fibroblasts (MEFs) increased survival and decreased apoptotic activation following DNA damage, relative to wild-type (WT) Ku70 despite having no impact on DNA repair efficiency. Furthermore, this mutation prevented the activation of a DDR, dependent upon Activating Transcription Factor 2 (ATF2). Since this residue was a serine, a common site for phosphorylation in the DDR, we hypothesized that this mutation was preventing a phosphorylation event. Indeed, a phosphomimetic substitution of S155 to aspartic acid (S155D) constitutively activated ATF2 and conferred a severe hypersensitivity to IR.

In the present study, we demonstrate that Ku70 S155 is indeed phosphorylated after IR, and show that constitutive expression of the phosphomimetic mutant Ku70 S155D induces a DDR marked by a constitutive activation of ATM and cell cycle arrest at both the G1/S and G2/M checkpoints. Additionally, we show that Ku70 S155D interacts with the Aurora B kinase and mediates the inhibition of its kinase activity. The interaction of WT Ku70 and Aurora B was detected following IR treatment, but not in absence of DNA damage. This suggests that Ku70 phosphorylation after IR at serine 155 mediates the interaction and inhibition of Aurora B, contributing to the activation of the DDR and cell cycle arrest.

## Results

### Ku70 Serine 155 is phosphorylated in response to DNA damage

We previously established a retroviral system using a murine stem cell virus construct (pMSCV) to re-express WT Ku70 and Ku70 mutants in immortalized Ku70-deficient mouse embryonic fibroblasts (Ku70^−/−^ MEFs)^26^. This method generates pools of Ku70 knockout (KO) MEFs that stably re-express Ku70 allowing the restoration of endogenous Ku80 expression and yielding a functional Ku dimer. Using this system, we determined that MEFs expressing Ku70 S155 amino acid substitutions exhibited altered survival following IR treatment^26^. A mutant Ku bearing a Ku70 S155 substitution to alanine (Ku70 S155A) enhanced survival following IR treatment, causing a DNA damage signaling defect and compromising the activation of apoptosis in response to DNA damage. In contrast, substitution to a phosphomimetic residue (S155D) conferred hypersensitivity to IR treatment. These analyses suggested that S155 is a phosphorylation site that is targeted for phosphorylation in response to DNA damage. To determine whether this site is phosphorylated following DNA damage, we conducted Mass Spectrometry (MS) analyses on Ku70 immunoprecipitated from nuclear extracts obtained from WT Ku70-expressing MEFs either untreated, or 30 minutes following a 40 Gy IR treatment. Chymotryptic digestion of immunoprecipitated Ku peptides revealed a peak corresponding to a peptide doubly phosphorylated at positions S155 and S162 in the irradiated sample, but not in the unirradiated control samples (Supplementary Figure 1A). To confirm these results, we repeated the analysis with extracts obtained from Ku70 S155A-expressing MEFs. In this case, only a mono-phosphorylated peptide was observed, consistent with the mutation preventing phosphorylation at S155 and being solely phosphorylated at S162 (Supplementary Figure 1B). Thus, we repeated the procedure with MEFs expressing a Ku70 mutant bearing an alanine substitution at position S162. Again, only one peak corresponding to the mono-phosphorylated peptide was observed, suggesting that this peptide was now only phosphorylated at the S155 position (Supplementary Figure 1C). Neither control samples obtained from Ku70 S155A nor S162A-expressing MEFs contained a peak corresponding to a peptide with phosphorylated S155. Overall these results suggest that Ku70 is phosphorylated at S155 after IR.

### Ku70 S155D induces cell cycle arrest

**Figure 1:**
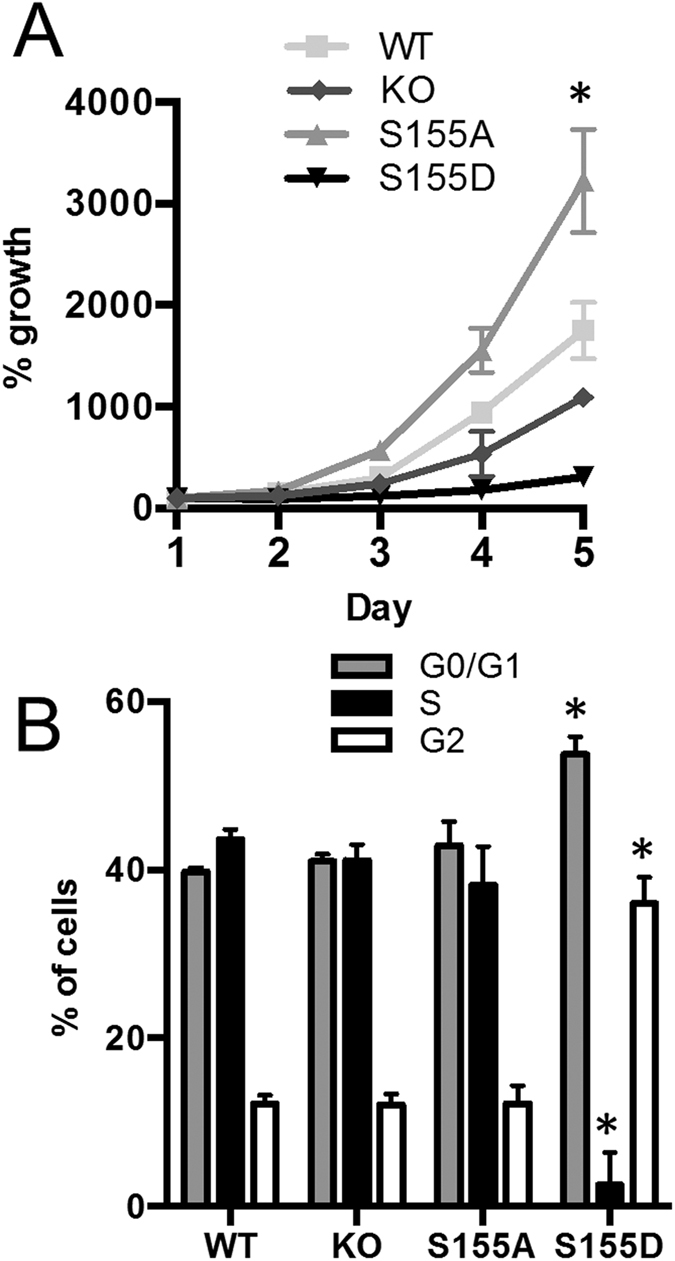
Expression of Ku70 S155D triggers cell cycle arrest. (**A**) Ku70 S155 mutants confer altered cell proliferation. Growth rates of Ku70^−/−^ MEFs expressing Ku70 WT, S155A, S155D or empty vector (KO) were assessed for 5 days. Data represents percent growth relative to Day 1 with error bars indicating SEM (*P < 0.05, all cell lines compared to WT, n = 3). Error bars are included for all data points but are not visible when smaller than symbol size. (**B**) Ku70 S155D cells arrest in G1 and G2. FACS analysis of DNA content and EdU incorporation in asynchronous Ku70^−/−^ MEFs expressing Ku70 WT, S155A, S155D or empty vector (KO) stained with propidium iodide and Anti-Edu Alexa 647. Average percentage of cells in each stage was determined by FlowJo cell cycle analysis in three separate experiments with error bars indicating SEM (*P < 0.01 S155D compared to all other cell lines, n = 3).

While the expression of the Ku70 S155D mutant in Ku70^−/−^ MEFs led to a strong hypersensitivity to IR^26^, we also noted that Ku70 S155D-expressing MEFs proliferated abnormally under standard culturing conditions. Growth rate analysis in the absence of any DNA damaging agent showed that expression of Ku70 S155D conferred a marked defect in proliferation, as these cells exhibited a 5.7-fold decrease in percent growth compared to Ku70^−/−^ MEFs re-expressing Ku WT by the fifth day of culture, and were even 3.6-fold slower than Ku70^−/−^ MEFs, which have been previously reported to have proliferation defects^27^ (Fig. 1A). In contrast, the Ku70 S155A-expressing MEFs proliferated faster than the WT counterparts, achieving a 1.8-fold increase over Ku70 WT MEFs by the fifth day of culture. In order to further analyze this proliferation defect, we compared the cell cycle profile of Ku70 WT, S155D, S155A cells and Ku70^−/−^ MEFs (Fig. 1B). Fluorescence activated cell sorting (FACS) analysis of Ku70 S155D-expressing MEFs showed that 53% of cells were in G1 phase, higher than the approximately 40% seen in either WT, KO or S155A-expressing MEFs. Similarly, 36% of Ku70 S155D-expressing MEFs were in the G2 phase, higher than the approximately 12% seen in the WT, S155A and Ku70^−/−^ cells. The largest difference was in S phase, where only 2.5% of S155D cells were found versus about 40% for the other three cell lines. Altogether this indicates that the Ku70 serine 155 residue regulates cellular proliferation and the expression of the phosphomimetic S155D mutation induces cell cycle arrest at both the G1/S and G2/M checkpoints.

**Figure 2:**
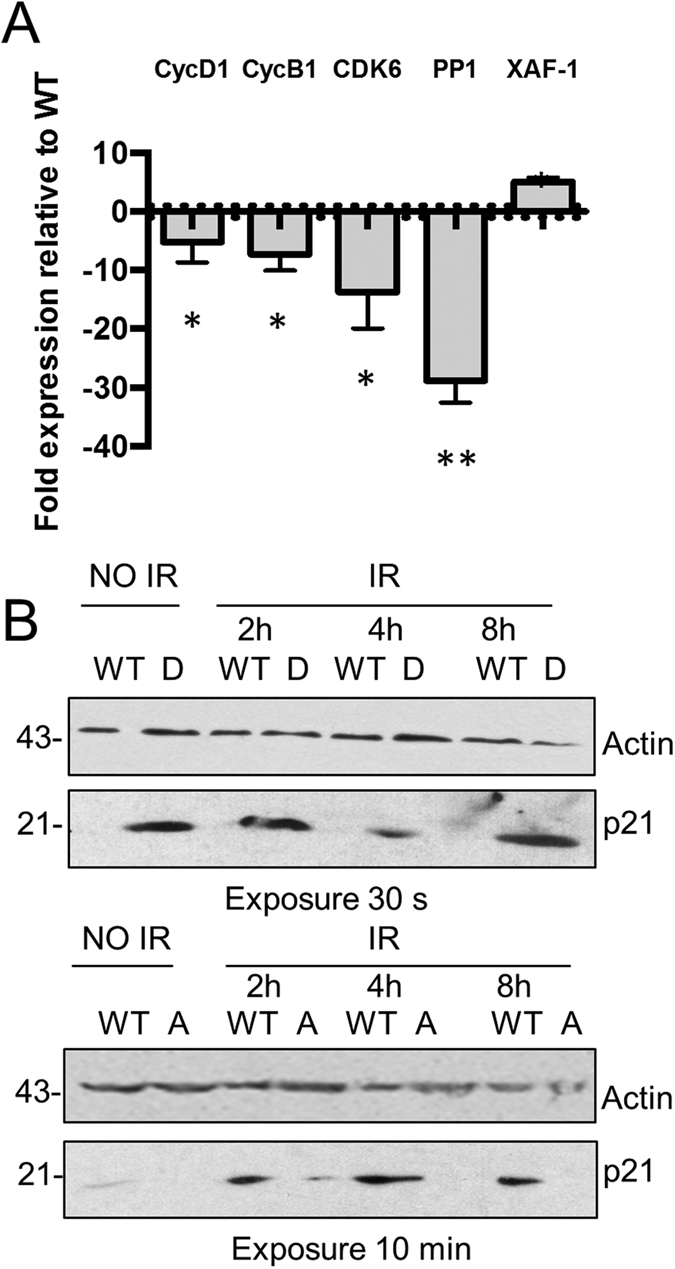
Ku70 S155D-expressing MEFs display altered expression of cell cycle-related factors. (**A**) RT-PCR analysis of Cyclin D, Cyclin B, CDK6, PP1 and XAF-1 gene expression. RNA samples from WT or S155D Ku70-expressing MEFs were analyzed by RT-qPCR using primer sets specific for the indicated genes. The fold change in gene expression of Ku70 S155D relative to Ku70 WT MEFs is shown, with error bars indicating SEM (**P < 0.01, *P < 0.05, n = 4). (**B**) Comparison of p21 expression in Ku70 WT and mutant MEFs. Representative western blot analysis of p21 in Ku70 WT and S155D expressing MEFs without IR (above) and p21 induction at the time points indicated after 10 Gy of IR in Ku70 WT and S155A MEFs (below). Similar amount of extracts were loaded on both gels (30 μg). Due to the strong expression of p21 in the Ku70 S155D MEFs, the top western blot required only a 30-second exposure, compared to a 10-minute exposure for the western blot below to obtain a similar intensity of p21 signal in response to DNA damage. Exposure time for the actin signal is similar for both western blots.

To analyze the global transcriptional changes that resulted in cell cycle arrest, an Affymetrix GeneChip was performed using RNA from Ku70^−/−^ MEFs expressing Ku70 WT and S155D. Several genes were either up- or downregulated in the Ku70 S155D-expressing MEFs relative to WT control (Supplementary Figure 2), and subsequently, some were validated by RT-qPCR (Fig. 2A). Cyclin D and CDK6, two proteins that form a complex to promote the progression through the G1/S checkpoint and Cyclin B, whose expression increases during the G2/M transition^28^, were downregulated in Ku70 S155D-expressing MEFs. Similarly, expression of Protein phosphatase 1 (PP1), a serine/threonine phosphatase, involved in the dephosphorylation of several targets during the DDR to resolve DNA damage checkpoints^29^, was downregulated in Ku70 S155D-expressing MEFs. Finally, the X-linked inhibitor of apoptosis-associated factor 1 (XAF-1), which regulates apoptosis and G2/M arrest^30^,^31^, was upregulated in Ku70 S155D MEFs. We also assessed the protein levels of p21, an inhibitor of cyclin D and B-CDK complexes which is upregulated in a p53 dependent manner following DNA damage and activates the G1 and G2 cell cycle checkpoints^32^,^33^. Western blot analysis showed that while Ku70 WT MEFs required IR treatment to induce expression of p21, Ku70 S155D cells displayed a marked upregulation of p21 in the absence of any treatment (Fig. 2B). Conversely, the Ku70 S155A MEFs failed to upregulate p21 levels, even after IR treatment. Overall, consistent with the observation that the expression of Ku70 S155D results in the arrest of cells in the G1 and G2 phases, this mutant alters the expression of cyclins known to regulate the progression through both the G1/S and G2/M checkpoints, and markedly upregulates the cell cycle inhibitor p21.

### Ku70 S155D induces a DNA damage response

**Figure 3:**
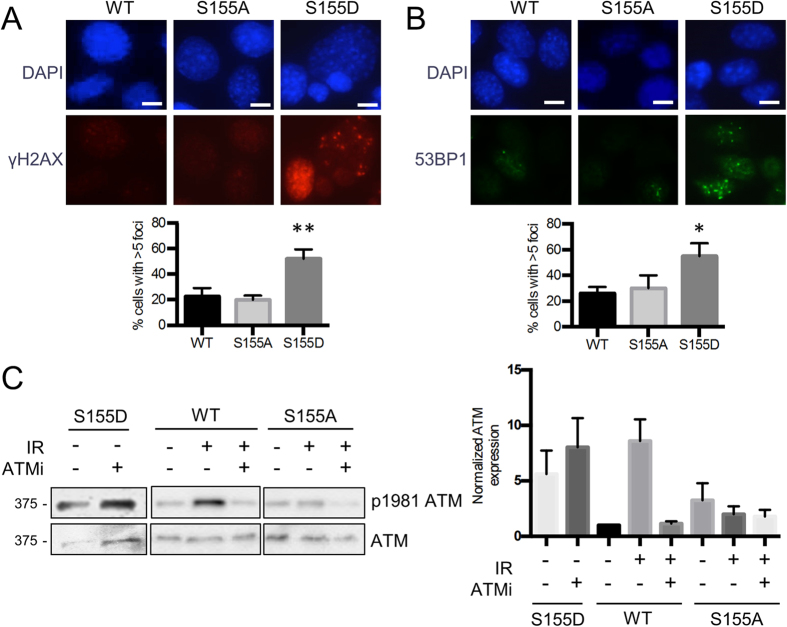
Ku70 S155D expression induces a DNA damage response in the absence of DNA damage. (**A**) Ku70 WT, S155D and S155A expressing MEFs were analyzed by immunofluorescence with a γ-H2AX antibody with representative pictures shown (left). Foci-containing cells were quantified and shown as an average of three experiments with error bars indicating SEM (right) (**P < 0.01, n = 3). Scale bars, 10 μm. Cells were scored positive when containing more than 5 foci. (**B**) Same procedure as in A, but the analysis was performed with a 53BP1 antibody (*P < 0.05, n = 3). (**C**) Western blot analysis of p-S1981 ATM in Ku70 S155D, WT and S155A expressing MEFs. Samples were either left untreated (control), collected 30 minutes following 4 Gy of IR (IR), or collected 30 minutes after 4 Gy of IR following a 1 hour incubation with 10 μM ATM inhibitor (ATMi). Quantification is shown (right panel) with error bars indicating SEM (n = 4).

We previously determined that a Ku70 S155A substitution decreased activation of apoptosis via an ATF2-dependent DDR pathway following IR treatment^26^. The altered proliferation of Ku70 S155 mutant MEFs however, was occurring in the absence of any DNA damaging agent, suggesting that mutation of Ku70 S155 had a direct effect on the cell cycle. To test this, we examined the presence of active DNA damage response markers following the expression of Ku70 S155 mutants. Strikingly, immunofluorescence (IF) analysis of Ku70 S155D MEFs showed significantly increased γ-H2AX and 53BP1 foci formation compared to both Ku70 WT and S155A MEFs, in the absence of any exogenous DNA damage (Fig. 3A,B). Furthermore, western blot analysis for activated ATM, marked by an auto-phosphorylation at serine 1981, showed increased levels (relative to WT) in untreated S155D MEFs, while S155A MEFs displayed very little activation of ATM after IR (Fig. 3C). Interestingly, pre-treatment of cells with the ATM inhibitor KU-55933^34^ prevented activation of ATM after IR in Ku70 WT, but had no impact on ATM activation in Ku70 S155D MEFs, suggesting that in these cells, ATM is constitutively phosphorylated to the levels reached in response to 4 Gy of IR (Fig. 3C) 34. We confirmed that Ku70 S155D expression does not affect the efficiency of DNA repair, nor induce detectable DNA breaks using both the neutral comet assay and the more sensitive alkaline comet assay (Supplementary Figure 3). Overall, these observations suggest that expression of the phosphomimetic Ku70 S155D constitutively activates an ATM-dependent DDR, in the absence of DNA damage.

### Ku70 S155D vWA domain is sufficient to induce cell cycle arrest

**Figure 4:**
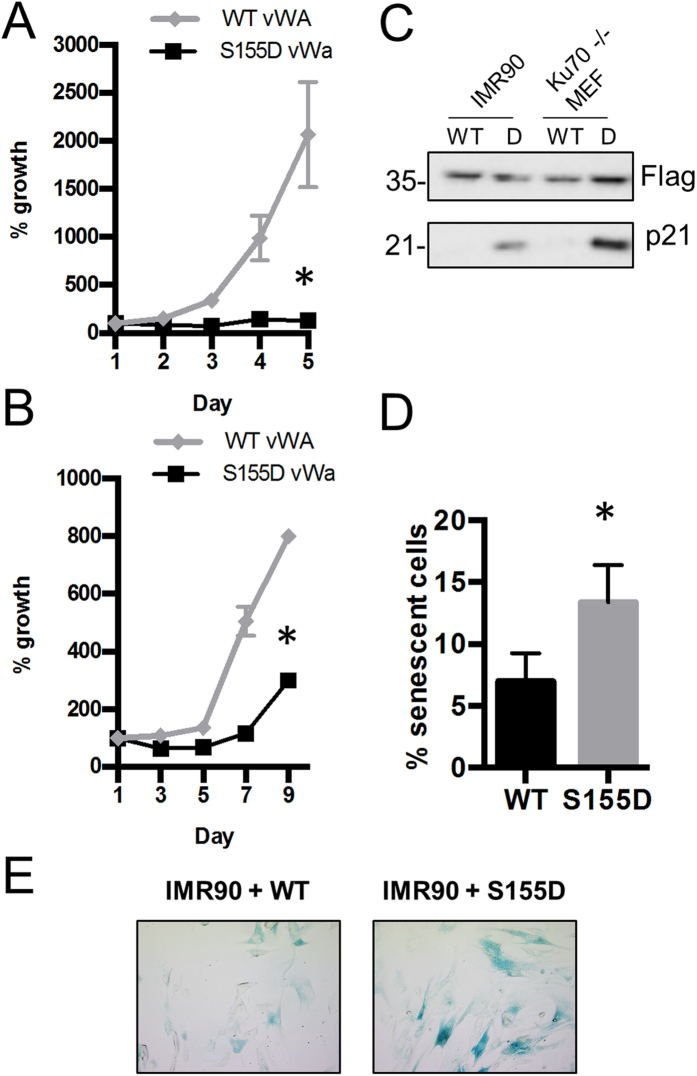
N-terminal Ku70 S155D domain is sufficient to induce a DDR and cell cycle arrest. (**A**) Growth rates of Ku70^−/−^ MEFs expressing FLAG-Ku70 WT or S155D vWA domain constructs were assessed for 5 days. Data represent percent growth relative to Day 1 with error bars indicating SEM (*P < 0.05 compared to WT, n = 3). Error bars are included for all data points but are not visible when smaller than symbol size. (**B**) Growth rates of IMR-90 cells were assayed as described above, for 10 days (n = 3). (**C**) Western blot analysis of p21 levels in IMR90 and Ku70^−/−^ MEFs expressing either FLAG-Ku70 WT or S155D vWA domain. (**D**) IMR-90 cells expressing FLAG-Ku70 WT or S155D vWA domain truncated constructs were stained with a solution containing X-gal (5-bromo-4-chloro-3-indolyl-β-D-galactopyranoside). The percentage of cells that stained blue, representing senescent cells, was quantified with error bars representing SEM (*P < 0.05, n = 3). (**E**) Representative pictures of ß-galactosidase staining of IMR-90 Ku70 vWA WT and S155D-expressing cells.

Ku is well characterized as a DNA binding protein functioning in both DNA repair via the NHEJ pathway and the protection of telomere ends^19^. In order to test whether the DNA binding activity of Ku was needed to produce the DDR induced by Ku70 S155D, we generated truncated Ku70 FLAG-tagged constructs containing only the vWA domain, either with the WT sequence or with the S155D substitution. The central ring domain is required for Ku70/Ku80 heterodimerization and DNA binding, so a truncated Ku70 vWA domain-only construct cannot fulfill either function^20^. Similar to the full-length (FL) Ku70 S155D construct, the expression of the S155D vWA domain in Ku70^−/−^ MEFs significantly decreased proliferation compared to the Ku70 WT vWA domain control (Fig. 4A). Also, consistent with what was observed previously with Ku70 S155D, we detected a marked upregulation of p21 levels in Ku70 S155D vWA MEFs (Fig. 4C). This suggests that recruitment of the Ku70 S155D mutant to DNA is not required to induce a DDR. These results were obtained in a Ku70 null background, so we sought to determine whether the Ku70 S155D mutation would have a dominant effect in a cell line that had normal Ku70 expression. Expression of the retroviral Ku70 vWA-only constructs in the human cell line IMR-90 produced similar results, with the Ku70 S155D vWA domain significantly reducing proliferation compared to the Ku70 WT vWA domain (Fig. 4B). Again, a marked upregulation of the p21 protein levels in the S155D vWA-expressing IMR-90 cells was observed compared to WT vWA (Fig. 4C). Additionally, a senescence phenotype was observed^4^, as a significantly increased level of β-galactosidase activity was found in S155D expressing cells relative to WT (Fig. 4D,E). Overall, the Ku70 S155D vWA domain does not require heterodimerization with Ku80 nor end-binding capabilities to induce cell cycle arrest and this phenotype is dominant to WT Ku.

### Ku70 S155D interacts with and inhibits Aurora B

Given that the Ku70 S155D vWA domain lacking both its DNA binding domain and the ability to heterodimerize with Ku80 induced cell cycle arrest, we hypothesized that this domain was acting by binding to another factor (s) and either constitutively activating or inhibiting its activity. A general screen for interacting factors was performed using biotin-conjugated peptides of the loop region surrounding S155 (aa 145 to 163) and comparing factors that bound to peptides containing either an alanine or aspartic phosphomimetic mutant at the serine 155 position. S155A or S155D peptides were incubated with MEF nuclear extracts from cells that were either untreated or subjected to 10 Gy of IR. Interacting factors were pulled down with streptavidin beads and identified by mass spectrometry.

**Figure 5:**
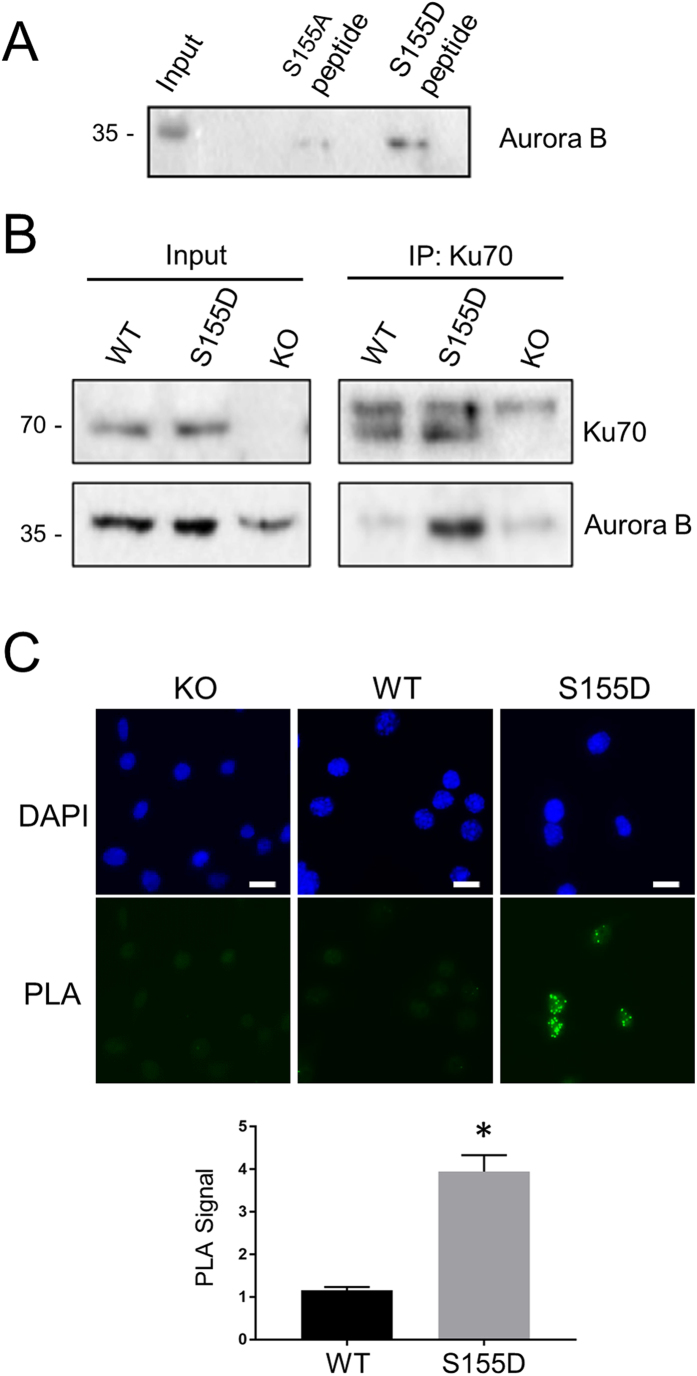
Ku70 S155D interacts with Aurora B. (**A**) Aurora B interacts with a Ku70 S155D peptide. Biotin-conjugated peptides of the 15 amino acids surrounding Ku70 S155 containing either a S155A or S155D substitution were used in a biotin-streptavidin pull-down with untreated WT Ku70 MEF extracts. Shown is a western blot analysis of Aurora B present in the samples pulled-down with the indicated peptides. (**B**) Interaction of Aurora B with Ku70 S155D. Extracts from Ku70^−/−^ MEFs stably expressing Ku70 WT, Ku70 S155D or empty vector were immunoprecipitated with a Ku70 antibody. The immunoprecipitates were analyzed by western blot with antibodies against Ku70 and Aurora B. (**C**) Interaction of Aurora B and Ku70 S155D using Proximity Ligation Assay (PLA). Ku70 KO, WT, and S155D expressing cells were fixed and incubated with antibodies to Ku70 and Aurora B and processed for PLA analysis. Shown are representative immunofluorescent images. Green dots represent the PLA signal, and the nuclei are stained with DAPI. Interactions were quantified by pixel density relative to the PLA signal in the Ku70 KO cells, and shown as an average of three experiments with error bars indicating SEM. (*P < 0.05, n = 3). Scale bars, 10 μm.

MS analysis identified Aurora B as a factor interacting with the S155D peptide in both control and IR-treated extracts (Supplementary Figure 4). The interaction of Aurora B with the S155D peptide was validated using pull-down of WT MEF extracts with S155 A/D peptides followed by western blot analysis (Fig. 5A). We then verified the interaction between Aurora B and full length Ku70 S155D. Ku70 was immunoprecipitated from nuclear extracts from Ku70^−/−^ MEFs (used as a control), and Ku70^−/−^ MEFs re-expressing WT Ku70 or Ku70 S155D. Endogenous Aurora B was efficiently co-immunoprecipitated with Ku70 S155D, indicating that Aurora B interacts with the phosphomimetic form of Ku70 (Fig. 5B). However, very little Aurora B was observed in the WT Ku70 immunoprecipitates, suggesting that phosphorylation of S155 is needed for interaction between the two proteins. Finally, we performed *in situ* Proximity Ligation Assay (PLA), a method that was developed to monitor interactions of endogenous proteins directly in individual cells^35^ to assess co-localization of Ku70 WT and the Ku70 S155D mutant with Aurora B (Fig. 5C). Only background PLA signal was observed in cells expressing Ku70 WT, but a robust signal was observed in Ku70 S155D-expressing cells, supporting the idea that complex formation is dependent on S155 phosphorylation.

Aurora B promotes the progression of the cell cycle through the G1/S, G2/M and mitotic checkpoints by the phosphorylation of several targets^5^. Interestingly, chemical inhibition of Aurora B results in cell cycle arrest and the activation of several DDR markers, notably γ-H2AX and 53BP1, phospho-ATM S1981 and upregulation of p21^15^,^16^. Thus, the effects of Ku70 S155D expression appeared to be similar to those reported in response to Aurora B inhibition, suggesting that interaction between Ku70 S155D and Aurora B could inhibit Aurora B.

**Figure 6:**
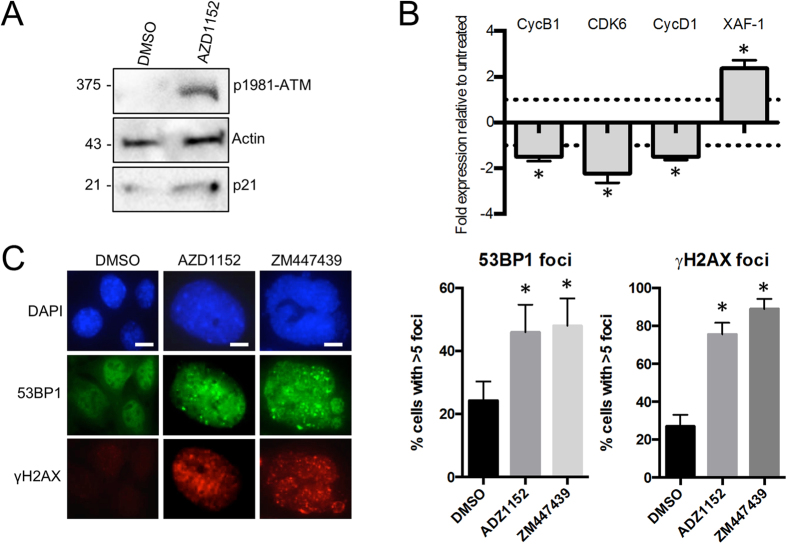
Aurora B chemical inhibition induces a DNA damage response. (**A**) Western blot analysis of p21 and pS1981-ATM levels in WT Ku70 MEFs treated for 48 h with either 20 nM of the Aurora B inhibitor AZD-1152 (+) or the DMSO vehicle control (−). (**B**) RT-PCR analysis of Cyclin B, CDK6, Cyclin D and XAF-1 in WT Ku70 MEFs treated with AZD-1152 as described in A, normalized to the DMSO vehicle control (*P < 0.05, n = 3). (**C**) Immunofluorescence analysis of γ-H2AX and 53BP1 foci formation in MEFs treated with AZD-1152 (20 nM), ZM447439 (50 nM) or DMSO vehicle control as described above. Cells were scored positive when containing more than 5 foci and the results shown with error bars indicating SEM (*P < 0.05, n = 3). Scale bars, 10 μm.

First we investigated whether inhibition of Aurora B in Ku70 WT MEFs produced the same cellular response as that observed in MEFs expressing the Ku70 S155D mutant. Treatment of WT MEFs with 20 nM of the Aurora B selective inhibitor AZD-1152^36^ triggered a marked upregulation of the cell cycle inhibitor p21 relative to control (vehicle-treated) samples as measured by western blot analysis (Fig. 6A). Next, we looked at the effect of Aurora B inhibition on the expression of the genes that we found deregulated in cells expressing Ku70 S155D. RT-PCR analysis of RNA extracted from Ku70 WT-expressing MEFs treated with AZD-1152 revealed, similar to what was observed in Ku70 S155D-expressing cells, Cyclin B, Cyclin D and CDK6 expression was significantly downregulated relative to the vehicle control-treated cells (Fig. 6B). Lastly, we investigated the effect of Aurora B inhibition on the activation of the DDR. Treatment of WT MEFs with Aurora B inhibitors AZD-1152 and ZM447439 significantly increased both 53BP1 and γ-H2AX foci (Fig. 6C), and AZD-1152 induced ATM activation as determined by pS1981-ATM levels (Fig. 6A). Thus, Aurora B inhibition induced a DNA damage response and transcriptional changes that mirrored those induced by Ku S155D expression.

**Figure 7:**
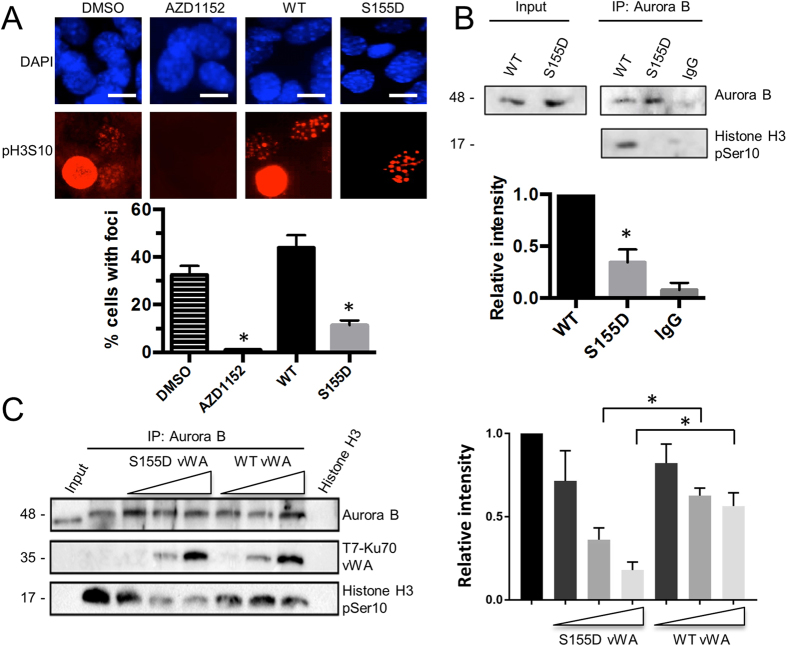
Ku70 S155D inhibits Aurora B kinase activity. (**A**) Immunofluorescence analysis of p-H3S10 in Ku70 WT and S155D-expressing MEFs. p-H3S10 was assessed in wild-type (Ku70^+/+^) MEFs treated with AZD-1152 (panel 2) or DMSO control (panel 1) and in Ku70^−/−^ MEFs expressing either Ku70 WT (panel 3) or Ku70 S155D (panel 4). p-H3S10 foci-positive cells were counted as a percentage of total cells. Top panels show representative pictures and quantifications are shown below with error bars indicating SEM (*P < 0.05, n = 3) for AZD-1152 relative to DMSO and S155D relative to WT. Scale bars, 10 μm. (**B**) Aurora B kinase activity assay. Ku70 WT or S155D-expressing MEF nuclear extracts were immunoprecipitated with an Aurora B antibody or IgG control. The immunoprecipitates were used to assay Histone H3 phosphorylation in an *in vitro* assay and analyzed by western blot with antibodies to phospho-H3S10 and Aurora B (top panel). Phospho-H3S10 western blot signal was quantified (bottom graph) and shown as an average with error bars indicating SEM (*P < 0.05, n = 3). (**C**) Aurora B kinase activity assay in the presence of increasing amounts of Ku70 S155D or WT vWA peptide. Ku70 WT-expressing MEF nuclear extracts were immunoprecipitated with an Aurora B antibody. The immunoprecipitates were incubated with 0, 1, 5, or 10 ng of bacterially expressed, T7 tagged-Ku70 S155D or WT vWA domains. Aurora B kinase activity was assessed using the *in vitro* kinase assay and analyzed by western blot with antibodies to phospho-H3S10, T7 tag, and Aurora B (left panel panel). Phospho-H3S10 western blot signal was quantified relative to immunoprecipitated Aurora B and is shown as an average with error bars indicating SEM (right panel) (*P < 0.05, n = 4).

Since Ku70 S155D-expressing MEFs display cell cycle arrest and activation of a DNA damage response, a phenotype observed in Aurora B inhibitor-treated MEFs, we hypothesized that Ku70 S155D interaction with Aurora B could be inhibiting the activity of Aurora B. To explore the impact of Ku70 S155D expression on Aurora B kinase activity, we monitored phosphorylation of histone H3 on serine 10 (H3S10), which is catalyzed by Aurora B^9^,^37^,^38^. Immunofluorescence analysis of phospho-H3S10 in MEFs expressing either WT or Ku70 S155D revealed that Ku70 S155D expression led to significantly decreased H3S10 phosphorylation compared to WT (11% of phospho-H3S10 foci-containing cells versus 43% in S155D) (Fig. 7A). However, although this result showed that the Ku70 S155D expression led to the loss of a specific Aurora B phosphorylation event, the loss of H3S10 phosphorylation would be expected to happen in cells experiencing prolonged cell cycle arrest and no longer undergoing mitosis. To further evaluate a direct effect of Ku70 S155D on Aurora B kinase activity, we carried out an *in vitro* assay in which endogenous Aurora B was immunoprecipitated from either Ku70 WT or S155D-expressing MEFs and incubated with purified histone H3 (Fig. 7B). Western blot analysis using an antibody directed against phosphorylated H3S10 detected phosphorylation with Aurora B immunoprecipitates from Ku70 WT-expressing MEFs in this assay. However, phosphorylation of H3S10 was significantly lower (2.9-fold) in immunoprecipitates from Ku70 S155D-expressing MEFs, suggesting that complex formation of Aurora B and Ku S155D has an inhibitory effect on Aurora B kinase activity. Since the Ku70 S155D vWA domain expression can induce cell cycle arrest, we tested whether the S155D vWA domain could also inhibit Aurora B. Kinase assays were conducted with Aurora B immunoprecipitated from Ku70 WT cells and incubated with bacterially expressed Ku70 vWA domain, either WT, or S155D (Fig. 7C). Some non-specific reduction of the kinase activity occurred upon addition of both peptides, however, the vWA peptide bearing the S155D mutation caused a significantly greater reduction of Aurora B kinase activity compared to the WT vWA, suggesting that the interaction of the S155D vWA domain with Aurora B has a specific inhibitory effect on its kinase activity.

### Ku70 and Aurora B interact following DNA damage

**Figure 8:**
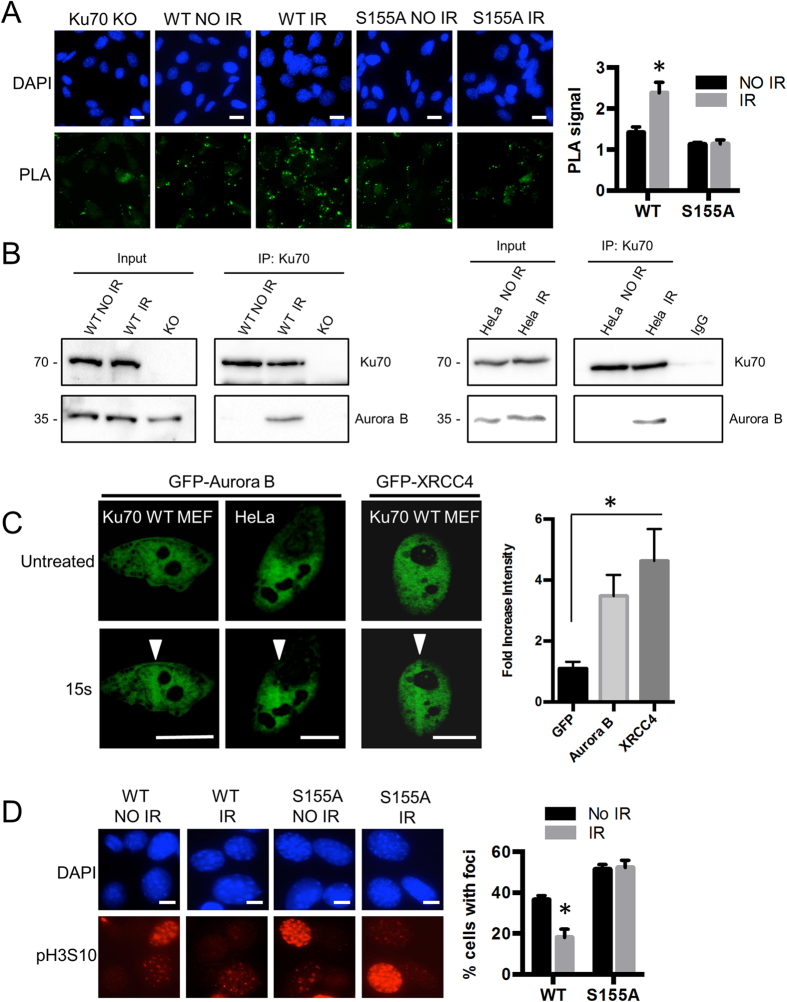
Ku70 interacts with Aurora B after IR and Aurora B inhibition after IR is dependent on Ku70 S155 phosphorylation. (**A**) Interaction of Aurora B and Ku after DNA damage using PLA. Ku70 WT and S155A-expressing MEFs were left untreated or were treated with 10 Gy of IR, fixed after 30-min and processed for PLA with antibodies to Ku70 and Aurora B analysis. Shown are representative immunofluorescent images. Green dots represent the PLA signal, the nuclei are stained with DAPI. Interactions were quantified by pixel density relative to the PLA signal in the Ku70 KO cells with error bars indicating SEM. (*P < 0.05, n = 3). Scale bars, 10 μm. (**B**) Aurora B co-immunoprecipitates with Ku70 after DNA damage. *Left*, Ku70^−/−^ MEFs expressing Ku70 WT (WT) or empty vector (KO) were subjected to IR treatment (40Gy) or left untreated (no IR) and extracts were prepared after 30 min incubation. Immunoprecipitation was performed with a Ku70 antibody and immunoprecipitates were analyzed by western blot with antibodies against Ku70 and Aurora B. *Right*, Hela cells treated with IR (40Gy) or left untreated (no IR) were processed as described above with a Ku70 antibody or IgG control. (**C**) GFP- Aurora B accumulates at the sites of laser microirradiation-induced DNA damage in MEFs and Hela cells. *Left*, GFP-Aurora B was transfected in WT MEFs and in Hela cells and GFP-Aurora B-expressing cells were subjected to laser microirradiation. Path of laser damage is shown with white arrow and time post treatment is indicated. For comparison, analysis was repeated with GFP-tagged NHEJ factor XRCC4. Scale bars, 10 μm. *Right*, quantifications of fluorescence intensity at the laser microirradiation sites. Graph shows fold increase in signal intensity of GFP (n = 3), GFP-Aurora B (n = 6) and GFP-XRCC4 (n = 7) 30 sec after microirradiation relative to the same site prior to irradiation. Error bars indicate SEM, *P < 0.05. (**D**) Immunofluorescence analysis of phospho-H3S10 in Ku70 WT and S155A-expressing MEFs either untreated, or irradiated with 10 Gy of IR and incubated for 2 hours. Foci-positive cells were counted as a percentage of total cells with error bars indicating SEM (*P < 0.05, n = 3). Scale bars, 10 μm.

Thus far, our results indicated that Ku70 S155 was phosphorylated in response to IR and that Ku70 containing the phosphomimetic substitution S155D interacted with and inhibited the activity of Aurora B. These data led us to speculate that *in vivo*, Ku70 phosphorylation after IR could function to inhibit the activity of Aurora B and prevent cell cycle progression, potentially leading to the activation of cell cycle checkpoints and senescence. In order to test this model, we sought to determine if Ku70 and Aurora B interact following IR treatment. Since the localization of Ku to DSBs is difficult to observe directly by microscopy as Ku does not form foci in response to IR^39^, we employed a proximity ligation assay (PLA). PLA was performed in Ku70 WT or S155A MEFs with antibodies directed against both Ku70 and Aurora B (Fig. 8A). In untreated samples, few dots were detected in either the Ku70 WT or S155A cells. However, in cells treated with 10 Gy of IR, a significant increase in the number of dots was observed in the Ku70 WT MEFs, while no change was detected in the Ku70 S155A MEFs. These results suggest that, while Ku70 and Aurora B do not associate in normally proliferating cells, a complex is formed following DNA damage. This interaction is dependent upon the phosphorylation of Ku70 at S155, as it is not observed in the S155A samples, where the residue is not phosphorylated. To substantiate this result, we performed co-immunoprecipitation analyses to assess Aurora B interaction with Ku following IR treatment (Fig. 8B). Aurora B was found to co-immunoprecipitate with Ku in both MEF and Hela IR-treated extracts, but not in control extracts, confirming that Ku-Aurora B complex formation is induced by DNA damage. We subsequently confirmed that Aurora B is recruited to DNA breaks, by assessing the recruitment of GFP-Aurora B at DSBs induced by laser microirradiation in both MEFs and Hela cells (Fig. 8C). In irradiated regions, GFP-Aurora B reached accumulation levels comparable to those of GFP-XRCC4, a key member of the NHEJ complex which recruitment to DSBs has previously been characterized^40^,^41^. Time-lapse imaging revealed similar kinetics of Aurora B recruitment in MEFs and Hela cells, with a maximal accumulation reached in about 20 sec following completion of laser damage, similar to what has been reported for the recruitment other factors at laser-induced DSBs^40^,^41^ (Supplementary Figure 5).

We next investigated the effect of Ku70 S155 mutations on Aurora B activity following IR, as measured by pH3S10 foci formation. In Ku70 WT MEFs, a decrease in H3S10 phosphorylation was observed 2 hours following IR suggesting a decrease in Aurora B activity (Fig. 8D). In Ku70 S155A MEFs, however, no significant difference in the pH3S10 levels was detected between control and IR samples suggesting that Aurora B activity was not reduced in these cells. This suggests that phosphorylation on Ku70 S155 in response to DNA damage functions to promote its interaction with Aurora B and results in inhibition of its kinase activity.

## Discussion

This study identifies serine 155 of Ku70 as a novel phosphorylation site following DNA damage. Through expression of a phosphomimetic S155D mutant, we demonstrated that this phosphorylation event induces a DDR and cell cycle arrest. We found that Ku phosphorylated at Ku70 S155 interacts with the Aurora B kinase and leads to inhibition of its activity, suggesting that the DNA damage signaling events activated by Ku70 S155D occur through Ku’s modulation of Aurora B kinase activity.

Expression of a phosphomimetic substitution at the serine 155 site into Ku70^−/−^ MEFs induces a potent DDR leading to cell cycle arrest and senescence. This is in contrast to what is observed with cells expressing Ku70 with an alanine substitution at the S155 site, which exhibit increased growth rate, increased survival and decreased activation of DNA damage markers after IR. These opposing phenotypes led us to hypothesize that S155 is modified and that the alanine substitution prevents a phosphorylation event that affects cell survival following DNA damage. Phosphorylated Ku has been observed in large scale and *in vitro* proteomic studies, in a number of different contexts and cell types. These include human Hela and K562 cancer cell lines, and upon mTOR-dependent signaling, stem cell differentiation and, interestingly, mitotic kinase inhibition^42^,^43^,^44^,^45^,^46^. However in many cases functional significance of this phosphorylation could not be demonstrated. For example, alanine substitutions of several residues phosphorylated by DNA-PK *in vitro* had no impact on NHEJ efficiency^47^,^48^. Interestingly, some of these same residues, located in the Ku70 N-terminus, were deemed essential for apoptotic activation in neurons^49^. Interestingly, recent reports have demonstrated that Ku phosphorylation can affect DNA repair, either by increasing DNA repair efficiency or by influencing the DSB repair pathway choice by inducing Ku’s dissociation from DNA in S phase to promote DNA repair by HR^50^,^51^. Here, we show that phosphorylation of Ku70 on serine 155 is a DNA damage-induced event and that Ku S155 modulates DNA damage signaling. The fact that S155 phosphorylation was not detected in previous proteomic studies could be due to low abundance of the pS155 peptide. We propose that S155 phosphorylation occurs on Ku molecules actively involved in NHEJ at the DNA break, and in the event of overwhelming damage that requires the activation of apoptosis or prolonged cell cycle arrest. This would result in very few phosphorylated Ku molecules relative to the total amount of this highly abundant protein. We optimized detection of this residue by treating cells with a high dose of IR and immunoprecipitating Ku70, but still observed the phosphorylated peptide in low abundance. Furthermore, the previous proteomics studies employed proteolytic enzymes other than chymotrypsin and could have been producing pS155 peptides without a composition favorable for MS detection. It should be noted that our results also indicate a probable phosphorylation at the S162 position, a site that has not been reported in these large-scale studies.

It remains to be determined which kinase is responsible for phosphorylating S155 as it does not fall within any canonical kinase motif. Possible candidates include ATM and DNA-PK_CS_, which recognize a loose substrate consensus motif, S/TQ^52^, but which have also been suggested to phosphorylate S/TXXQ sequences fulfilled by the SDVQ sequence of Ku70^53^. Many other possibilities exist however, due to the abundance of serine/threonine kinases involved in the DDR. Indeed, proteomic data have implicated the kinases Chk1, CDK2 and Polo-like kinase in Ku phosphorylation^44^,^54^,^55^.

While Ku70 S155D-expressing cells exhibited growth arrest, those expressing Ku70 S155A grew faster than their Ku70 wild-type counterparts. This was somewhat surprising, so to determine whether there were changes in gene expression in Ku70 S155A cells that could explain this phenomenon, we looked into our previous microarray analyses^19^ to determine if Ku70 S155A expression influenced gene expression in absence of DNA damage. Interestingly, we noticed that several genes regulating cell proliferation and cell cycle which expression is affected by Ku70 S155D were also affected in Ku70 S155A cells in absence of DNA damage, but in the reverse manner. For instance, the expression of the Id genes (Id1/2/3) which promote cell proliferation were found to be slightly increased in S155A cells, whereas they were decreased in S155D cells. Reversely, Xaf-1, Ddit3 and ATF3 which promote cell cycle arrest and are found up-regulated in S155D cells, were found downregulated in S155A cells (data not shown). This indicates that the S155 residue influences cell proliferation via an effect on gene expression that modulates cell cycle. One possibility to explain this is that endogenous DNA damage causes some Ku S155 phosphorylation events that may be able to slow down the cell cycle. This occurs in Ku70 WT cells, but does not happen in the S155A-expressing MEFs, resulting in faster growth. Thus, the S155A mutant has the reverse effect of the S155D mutant, and facilitates cell cycle progression.

The expression of Ku70 S155D induced a DDR marked by H2AX phosphorylation, 53BP1 foci accumulation and activation of ATM. This phenotype correlates with the phenotypes observed in cells treated with Aurora B inhibitors and suggests that the effects of Ku S155D are conferred, at least in part, through its inhibition of Aurora B activity. Studies using the broad Aurora kinase family inhibitors MLN8054, MK-0457, and VE-465, as well as our own study employing the specific Aurora B inhibitors AZD-1152 and ZM447439, observed that these compounds induce γ-H2AX and 53BP1 foci formation, and the activation of ATM signaling, as measured by ATM phosphorylation^15^,^16^. AZD-1552 treatment has been further shown to slow growth rate, induce cell cycle arrest and senescence, and increase sensitivity to IR, all phenotypes observed following Ku70 S155D expression^12^,^13^,^14^,^56^. We also observed some enlarged nuclei in Ku70 S155D-expressing MEFs (Fig. 3A), a phenomenon indicative of failed cytokinesis and polyploidy, another common consequence of Aurora B inhibition^15^,^57^.

While Ku70 S155D triggered constitutive activation of ATM, cells expressing Ku70 S155A showed defective ATM activation in response to DNA damage. This is consistent with our previous observations that preventing phosphorylation at S155 results in reduced DDR activation^26^ and suggests that Ku S155 phosphorylation functions to enhance or maintain DNA damage signaling. Ku and ATM share interactions with several ATM regulators but many proteins and pathways contribute to ATM activation^19^,^58^. It is unknown at present whether Ku70 S155 phosphorylation modulates ATM activation directly, or indirectly through inhibition of Aurora B and/or through an effect on an ATM regulator.

Perhaps the most striking effect of Ku S155D expression is its ability to strongly activate p21. p21 gene (Cdkn1a) expression was increased in S155D cells (see Supplementary Figure 2) and p21 protein levels were markedly upregulated. In contrast, in cells expressing Ku70 S155A, p21 induction was severely attenuated in response to DNA damage. p21 is activated through ATM, so enhanced activation of ATM in S155D cells could contribute to its upregulation. In addition, Aurora B has been shown to repress p21 expression and Aurora B downregulation or inhibition was shown to result in p21 upregulation^59^,^60^. Again, this is consistent with an inhibitory role for Ku70 S155D on Aurora B activity leading to p21 activation, and with a lack of repression of Aurora B in Ku70 S155A cells, preventing p21 induction in response to DNA damage. Interestingly, Kumari *et al.* revealed that Aurora B inhibition activates p38 MAP kinase, which in turns promotes p21 induction by promoting transcriptional elongation of p21 transcripts^61^. p38 is known to activate the transcription factor ATF2, which is also found strongly activated in Ku S155D MEFs^26^. In addition, the expression of ATF3, a downstream target of ATF2 that is upregulated in the Ku70 S155D-expressing cells (Supplementary Figure 2) is increased through Aurora B inhibition in a p38-dependent manner^62^,^63^. Altogether, the similarities between the effects caused by Aurora B inhibition and Ku70 S155D expression are consistent with the inhibition of Aurora B by Ku70 S155D.

Expression of a truncated Ku70 S155D comprising only the vWA domain triggered a similar growth defect in both Ku70^−/−^ MEFs and in the cell line IMR90 which are non-transformed human lung cells that express normal levels of Ku. This demonstrates that the Ku70 S155D substitution exerts a dominant-negative effect, and that Ku70 S155 regulation is not specific to a particular cell line, although the amplitude of the response may vary depending on cell type. Moreover, it suggests that Ku70 S155D can exert its effects in absence of DNA binding. Indeed, the Ku70 vWA peptide lacks both heterodimerization and end-binding activities. However, we cannot exclude that it interacts with telomeres. How Ku binds to telomeres, whether it is through direct binding to telomeric DNA or through telomere protein interactions is still a matter of debate^19^. Interestingly, residues in Ku70 vWA domain Helix 5 have been suggested to interact with the shelterin protein TRF2, however, whether this interaction is sufficient to recruit Ku to telomeres has not been investigated^64^.

The interaction between Aurora B and Ku was dependent on phosphorylation at S155. We showed that while Ku70 and Aurora B do not interact in normally proliferating cells, Aurora B does associate with the phospho-form of Ku70, and they form a complex following DNA damage. Importantly, we found that Aurora B inhibition was dependent on Ku70 S155 phosphorylation as it was not observed in Ku S155A-expressing cells, which suggests that the interaction of Ku pS155 with Aurora B is required for its inhibition. The full activation of Aurora B is achieved through conformational changes induced by the binding of protein cofactors^65^. There are a number of known activators of Aurora B, well characterized examples being the members of the chromosomal passenger complex (CPC), and this diversity allows for the specific targeting and local activation of Aurora B to different chromosomal structures^66^. Similarly, inhibition of Aurora B is mediated through protein-protein interactions, including phosphatases PP1, PP2A and the checkpoint protein BubR1^66^. We propose that Ku, interacting with Aurora B either directly or through association with members of the Aurora B complex, is an inhibitory cofactor following DNA damage. Thereby, the phosphomimetic S155D Ku70 mutant acts as a dominant negative, by constitutively binding and inhibiting Aurora B, either through direct hindrance of its catalytic activity or by precluding the interactions with other activating cofactors.

There is a body of evidence linking DNA repair proteins to the inhibition of Aurora B kinase activity. Similar to what we observed with Ku70 S155D, the alternative end-joining DSB repair protein PARP-1 was demonstrated to interact with and inhibit Aurora B kinase activity, in this case as a result of direct ribosylation of Aurora B^67^. The HR proteins BRCA2/BARD1 were also shown to negatively regulate Aurora B by promoting its degradation^68^. Together with our results, this suggests that several mechanisms involving factors from the three different DSB repair pathways converge to inhibit Aurora B activity in response to DNA damage. Recruitment of Aurora B at the site of DNA damage may serve to ensure its inhibition to allow sustained cell cycle arrest to repair damaged DNA.

Overall, our results suggest that upon the introduction of DNA damage, unphosphorylated Ku is recruited to DNA breaks where it promotes the assembly of the NHEJ DNA repair complex. We propose that Ku is phosphorylated at S155 under conditions of overwhelming damage or when the DNA break is too complex for proper repair. This event ensures the sustained activation of the DDR and Aurora B inhibition to reinforce cell cycle arrest, providing time to complete DNA repair. If repair cannot be completed, the persistence of Ku70 S155 phosphorylation could contribute to senescence or activation of apoptotic pathways. The role of Aurora B in the DDR is still poorly understood, however, it has previously been linked to prominent regulators of the DDR, such as the Repo-Man/PP1 complex and ATM^69^,^70^,^71^. Our data provide additional evidence that Aurora B plays an important role in the response of cells to DNA damage. Our study also reinforces the notion that Ku’s presence at the DNA break not only serves to recruit the NHEJ machinery, but also functions to relay signals to the DDR to modulate cellular responses, presumably as a function of DNA repair completion.

## Materials and Methods

### Plasmid expression constructs

Ku70 WT, S155D and S155A pMSCVpuro constructs were previously described^26^. Ku70 WT, S155D vWA-FLAG pMSCVpuro constructs were produced by subcloning the XhoI and EcoRV fragment (aa 1–250) of full length Ku70 into the pMSCVpuro vector, and then inserting a FLAG tag by oligonucleotide annealing (Sigma-Aldrich, Oakville, ON). Similarly, the Ku70 WT and S155D vWA fragment was sublconed in pET-28-a (Novagen, Millipore) for bacterial expression. Ku70-HA constructs were obtained by adding an HA tag at the C-terminus of Ku70 by PCR. GFP-Aurora B was a gift from Gerd Pfeifer (Beckman Research Institute, City of Hope). GFP-XRCC4 was previously described^72^ and was kindly provided by Akira Yasui (Tohoku University, Sendai, Japan).

### Cell culture and treatments

Immortalized Ku70^−/−^ MEFs were obtained from S. Matsuyama (Case Western, Cleveland). Hela and IMR-90 cells were obtained from ATCC. All cells were cultured in high glucose Dulbecco’s modified Eagle’s medium (DMEM) supplemented with 10% fetal bovine serum (FBS) at 37 °C in 5% CO_2_. Ku70^−/−^ MEFs stably re-expressing Ku70 WT and mutants were generated by infecting Ku70^−/−^ MEFs with recombinant MSCV retrovirus followed by puromycin selection as previously described^26^. To assess proliferation rates, the Ku expressing MEFs were seeded in triplicate in 6-well dishes. At each time point cells were trypsinized, counted using a hemocytometer, and the mean number of cells was determined. Percent growth was obtained by dividing the number of cells at each time point by the number of cells at day 1. For irradiation experiments, cells were plated the night before at 50–70% confluency. Irradiations were performed with a Faxitron RX-650 at a dose rate of 1.42 Gy/min (10 Gy treatment) or 3.8 Gy/min (40 Gy treatment). For ATM inhibitor treatments, MEFs were incubated with 10 μM of KU-55933 (Selleck Chemicals, Houston, TX) for 1 hour prior to 4 Gy of irradiation. For Aurora B inhibitor treatments, MEFs were incubated with 20 nM of AZD-1152 and 50 nM ZM447439 (Sigma-Aldrich) for 48 hours.

### Extracts, Immunoprecipitation and Western Blot analyses

Nuclear Extracts were prepared as described previously^73^. For co-immunopreciptation experiments, extracts were adjusted to 0.15% NP-40 and 100 mM KCl, and incubated at 4 °C with either Ku70 antibody (N3H10, Santa Cruz, Santa Cruz, CA), or Ku80 antibody (C-20, Santa Cruz). Immunoprecipitates were isolated with Pierce Protein G magnetic beads (ThermoFisher Scientific, Rockford, IL). For Western blot analysis, extracts were resolved by SDS-PAGE, transferred onto a PVDF (Polyvinylidene fluoride) membrane and hybridized with the following antibodies: β-actin (I-19, Santa Cruz), Ku70 (N3H10, Santa Cruz), Ku80 (C-20, Santa Cruz), HA (HA-7, Sigma), p21 (C-19, Santa Cruz), phospho-serine 1981 ATM (Pierce, ThermoFisher Scientific), ATM (Pierce, ThermoFisher Scientific), phospho-serine 10 Histone H3 (Cell Signaling, Danvers, MA), Aurora B (H-75, Santa Cruz), FLAG (Sigma-Aldrich). The blots were developed using the Clarity Western ECL substrate (Bio-rad, Hercules, CA) and imaged on the Molecular Imager^®^ ChemiDocTM XRS system (Bio-Rad). Quantifications were performed using Image Lab software (Bio-Rad).

### Aurora B kinase assay

Immunoprecipitation was performed as described above with the Aurora B antibody (H-75, Santa Cruz). Immunoprecipitates were resuspended in kinase buffer (20 mM Tris-HCl, 20 mM KCl, 20 mM MgCl_2_, 0.4 μM ATP, 0.4 mM DTT) and incubated with 1 μg of purified Histone H3.1 (New England Biolabs) for 1 hour at 37 °C. The Ku70 WT and S155D vWA peptides were expressed in bacteria according to standard protocols. vWa domain-containing bacterial extracts were added to the immunoprecipitates and incubated for 30 min on ice before adding kinase buffer and Histone H3.1. Phosphorylation was detected by western blot with a phospho-H3S10 antibody (Cell Signaling).

### β-Galactosidase senescence assay

IMR-90 Cells were plated on 35 mm dishes or on glass coverslips in 24-well dishes. Cells were washed three times with Phosphate Buffer Saline (PBS) and fixed with 4% paraformaldehyde for 15 min at 4 °C. The staining solution (40 mM citric acid/Na phosphate buffer, 5 mM K_4_[Fe(CN)6] 3H_2_O, 5 mM K_3_[Fe(CN)6], 150 mM NaCl, 2 mM MgCl_2_, 1 mg ml-1 X-Gal (Bioshop Canada Inc, Burlington, ON) in DMSO, pH 6.0) was added to each dish and incubated for 12–16 h at 37 °C. Cells were viewed by bright field microscopy. Pictures were taken and blue cells were counted as a percentage of total cells.

### Reverse transcriptase PCR (RT-PCR)

Total RNA was isolated using the Qiagen RNeasy RNA extraction kit according to the manufacturer’s protocol and stored in aliquots frozen at −80 °C. RNA was quantified using a AstraLyra VIS/NIR Spectrometer (AstraNet, Cambridge, UK). The RNA (2 μg) was reverse transcribed with the Superscript II cDNA kit (Invitrogen, ThermoFisher Scientific) according to the manufacturer’s protocol. Quantitative PCR was performed using Bio-Rad MyiQ single-color real-time PCR detection and the Bio-Rad IQ SYBR green mix. The relative quantification of specific gene expression was determined by the ΔΔCT method, with the target gene threshold cycle (CT) values normalized to that of the beta-2-microglobulin control. Primers are listed in Supplementary Materials and Methods.

### Cell Cycle Analysis by Fluorescence-activated Cell Sorter (FACS)

Cells were incubated with 10 μM EdU for 1 hour then collected by trypsinization at a final concentration of 1 × 10^7^ cells/ml. The samples were processed using the EdU Click-It Alexa 647 Flow cytometry kit (Invitrogen) then stained in 0.1% Triton X-100 (Sigma-Aldrich), 0.2 mg/mL RNase A (Sigma-Aldrich), and 20 ug/mL of propidium iodide (Sigma-Aldrich). Cell cycle FACS was performed on a Calibur II (BD Biosciences, Mississauga, ON) and 50,000 gated events were measured per sample. Cell cycle modeling and statistics were performed on FlowJo software. All procedures were performed at the London Regional Flow Cytometry Facility.

### Immunofluorescence

Cells were seeded at 60–80% confluence on 10 mm glass coverslips, washed in cold PBS and fixed in 3% paraformaldehyde. Cells were permeabilized in 0.5% Triton-X and blocked in 5% FBS, followed by incubation with primary antibodies: phospho-serine 139 H2AX antibody (Abcam, Cambridge, UK), phospho-serine 1981 ATM (Pierce), Histone H3 phospho-serine 10 (Cell Signaling), 53BP1 (Pierce) and then with AlexaFluor 488/555/647 secondary antibodies (Invitrogen). All coverslips were mounted onto glass slides using ProLong Gold containing DAPI (Invitrogen). Cell pictures were taken with an Olympus BX51 microscope at 40x magnification using the Image-Pro Plus software (Media Cybernetics, Inc., Bethesda, MD). For γ-H2AX, 53BP1 and phospho-H3S10 analyses, cells were scored for the presence of foci, with DAPI nuclei staining used for total cell count. Foci-containing cells were quantified as a percentage of total cells, and approximately 500 cells were counted for each experimental condition per experiment.

The *in situ* proximity ligation assay (PLA) was performed with the Duolink (Olink Biosciences, Uppsala, Sweden) kit as per manufacturer instructions with minor modifications. MEFs were plated at 50–70% confluency onto 10 mm glass coverslips containing a hydrophobic wax boundary. Coverslips were fixed and permeabilized as described above and incubated overnight with Ku70 (Santa Cruz) and Aurora B (Santa Cruz) primary antibodies. Immunofluorescence images were obtained as described above. DAPI nuclei staining was used for total cell count and PLA signal was quantified by pixel density measurement in Image J software. The pixel density of the PLA was normalized to total cell count to obtain a mean PLA signal per cell. These measurements were then normalized to the background signal obtained in Ku70^−/−^ MEFs control samples.

### Laser Microirradiation

Ku70 knockout MEFs expressing wild-type Ku70 and HeLa cells were grown in 6-well plates and transfected with GFP-Aurora B or GFP-XRCC4 using jetPRIME transfection reagent (Polyplus Transfection). Twenty four hours after transfection, cells were sensitized with 1 μg/mL Hoechst 33342 (Life Technologies) for 15 minutes. Coverslips were then transferred to a Chamlide CMS magnetic chamber with fresh media and imaged with a Zeiss Axiovert 200 Motorized LSM 510 META NLO confocal microscope with a 63X Plan-Apochromat objective and 37 °C heated stage. Cells were microirradiated in defined 0.5 μm-wide rectangular regions with a 750 nm titanium-sapphire laser (Coherent) coupled to the microscope and set at 20% power with 20 iterations. A 488 nm Argon laser (Zeiss) with a 505 nm long-pass filter was used to image cells before and after microirradiation. Following microirradiation, images were acquired continuously, with an imaging time of 15.8 s. For quantitative analysis of protein recruitment, fluorescence intensities within the microirradiated regions of each cell were measured before and after laser damage using ImageJ 1.48v software (ImageJ, US National Institutes of Health, Bethesda, Maryland, USA). To correct for non-specific photobleaching, the fluorescence intensity of an untreated nuclear region was subtracted from each microirradiated region’s intensity before and after damage as described in Uematsu *et al.*^41^. The ratio of the corrected fluorescence intensity after treatment to the corrected fluorescence intensity before treatment was then calculated. Microirradiation data was analyzed using GraphPad Prism Version 6.05 for Windows (GraphPad Software, La Jolla, CA, USA).

### Statistical analyses

Differences between multiple groups was determined by an analysis of variance (ANOVA) and differences between two groups was determined by an unpaired two tail t-test. Results were considered significant when *P* < 0.05. Data were analyzed using GraphPad Prism Version 6.05 for Windows (GraphPad Software, La Jolla, CA, USA).

## Additional Information

**How to cite this article**: Fell, V. L. *et al.* Ku70 Serine 155 mediates Aurora B inhibition and activation of the DNA damage response. *Sci. Rep.* **6**, 37194; doi: 10.1038/srep37194 (2016).

**Publisher’s note**: Springer Nature remains neutral with regard to jurisdictional claims in published maps and institutional affiliations.

## Supplementary Material

Supplementary Information
