# Supplementary material for: Ku70 Serine 155 mediates Aurora B inhibition and activation of the DNA damage response
Source: Sci Rep. 2016 Nov 16;6:37194. doi: 10.1038/srep37194 (PMC5111114; doi:10.1038/srep37194)
Supplement: Supplementary Information [file srep37194-s1.pdf]

SUPPLEMENTARY INFORMATION

accompanying the manuscript entitled

Ku70 Serine 155 mediates Aurora B inhibition and activation of the DNA damage response

Victoria L. Fell, Elizabeth A. Walden, Sarah M. Hoffer, Stephanie R. Rogers, Amelia S. Aitken, Louisa M. Salemi and Caroline Schild-Poulter*

CONTENT:

Supplementary Figures 1-5

Supplementary Matherial and Methods

Supplementary Figure 1. Identification of S155 phosphorylation. (**A**) Experimental masses (m/z) obtained from MALDI analysis of Ku70 immunoprecipitated from both unirradiated and 30 minutes following a 40 Gy IR treatment WT Ku70 MEF. Samples were matched to theoretical masses obtained from MS-Digest. Peptides including Serine 155 highlighted in yellow with amino acid modifications are indicated. (**B**) Analysis as in A, with MEFs expressing Ku70 S155A. (**C**) Analysis as in B, with MEFs expressing Ku70 S162A.

A

WT Ku70 IR sample

Experimental (m/z)	Theoretical (m/z)	S/T Modification	Sequence
513.30072	513.2854		(D)LMHL(K)
524.16913	524.1843		(F)QDMM(G)
665.3548	665.3505		(D)TGIFLD(L)
668.36426	668.3436	1Oxidation	(D)VQFKM(S)
708.3714	708.3675		(D)KNSVNF(K)
708.3714	708.3675		(N)SVNFKN(I)
708.3714	708.3675		(L)RDTGIF(L)
723.36462	723.3494		(W)VC(Carbamidomethyl)ANLF(S)
741.39362	741.3964		(F)LDLMHL(K)
762.37238	762.3603		(L)WVC(Carbamidomethyl)ANL(F)
1077.5253	1077.5034		(L)SEVLWVC(Carbamidomethyl)AN(L)
1144.52	1144.4654		(L)FTNEDNPHGN(D)
1175.6154	1175.6129	1Oxidation	(D)TGIFLDLMHL(K)
1338.7152	1338.774		(E)LDNPGAKRILEL(D)
1353.7043	1353.7485		(L)QELDNPGAKRIL(E)
1401.6849	1401.6645		(F)YGTEKDKNSVNF(K)
1485.7487	1485.6831		(L)WVC(Carbamidomethyl)ANLFSDVQF(K)
1505.7894	1505.8079		(F)KMSHKRIMLFTN(E)
1520.6781	1520.6491	1Phospho	(D)YSLSEVLWVC(Carbamidomethyl)AN(L)
1522.7723	1522.8475	1Oxidation	(F)KMSHKRIMLFTN(E)
1580.8147	1580.705		(M)MGHGSDYSLSEVLW(V)
1594.8523	1594.8085		(D)QFKGQQGQKRFQD(M)
1597.8193	1597.7574	1Oxidation	(F)KGQQGQKRFQDMM(G)
1597.8193	1597.8107	1Phospho	(D)VQFKMSHKRIML(F)
1602.8799	1602.8139	1Phospho	(-)YISKIISSDRDLL(A)
1629.8309	1629.8005	2Oxidation 1Phospho	(D)VQFKMSHKRIML(F)
1769.9015	1769.8826	1Oxidation	(L)FSDVQFKMSHKRIM(L)
1895.8555	1895.8309	1Oxidation 2Phospho	(F)SDVQFKMSHKRIML(F)
1911.9117	1911.8258	2Oxidation 2Phospho	(F)SDVQFKMSHKRIML(F)
1962.988	1962.933	1Oxidation 1Phospho	(L)FSDVQFKMSHKRIML(F)
1962.988	1962.933	1Oxidation 1Phospho	(F)SDVQFKMSHKRIMLF(T)
1962.988	1962.933	1Oxidation 1Phospho	(N)LFSDVQFKMSHKRIM(L)

WT NO IR Sample

Experimental (m/z)	Theoretical (m/z)	S/T Modification	Sequence
513.30206	513.2854		(D)LMHL(K)
516.33014	516.314		(D)RDLL(A)
524.17188	524.1843		(F)QDMM(G)
598.31818	598.3235		(L)AVVFY(G)
623.33002	623.3035		(M)LFTNE(D)
644.3501	644.3072	1Oxidation	(L)DLMHL(K)
723.38635	723.3494		(W)VC(Carbamidomethyl)ANLF(S)
792.27747	792.2811	1Phospho	(F)YGTEKD(K)
855.45392	855.4247		(N)LFSDVQF(K)
996.55414	996.5037		(D)YSLSEVLW(V)
996.55414	996.5659	1Oxidation	(D)LMHLKKPGG(-)
1014.5139	1014.502	1Phospho	(D)LRDTGIFL(D)
1076.5718	1076.596		(F)KGQQGQKRF(Q)
1133.6256	1133.6161		(Y)ISKIISSDRD(L)
1142.5447	1142.521	2Oxidation 1Phospho	(F)KMSHKRIM(L)
1157.5491	1157.4697	1Phospho	(L)SEVLWVC(Carbamidomethyl)AN(L)
1175.6304	1175.6129	1Oxidation	(D)TGIFLDLMHL(K)
1193.5835	1193.6273		(E)KDKNSVNFKN(I)
1241.615	1241.6412	1Phospho	(D)LRDTGIFLDL(M)
1338.726	1338.774		(E)LDNPGAKRILEL(D)
1353.7074	1353.7485		(L)QELDNPGAKRIL(E)
1398.666	1398.6849	1Phospho	(D)SAKASRARTKAGD(L)
1401.681	1401.6645		(F)YGTEKDKNSVNF(K)
1431.7795	1431.8027		(D)SAKASRARTKAGDL(R)
1505.7802	1505.8079		(F)KMSHKRIMLFTN(E)
1520.6736	1520.6491	1Phospho	(D)YSLSEVLWVC(Carbamidomethyl)AN(L)
1522.7598	1522.8475	1Oxidation	(F)KMSHKRIMLFTN(E)
1594.8107	1594.8085		(D)QFKGQQGQKRFQD(M)
1597.8033	1597.7574	1Oxidation	(F)KGQQGQKRFQDMM(G)
1597.8033	1597.8107	1Phospho	(D)VQFKMSHKRIML(F)
1769.9053	1769.8826	1Oxidation	(L)FSDVQFKMSHKRIM(L)
1962.9733	1962.933	1Oxidation 1Phospho	(L)FSDVQFKMSHKRIML(F)
1962.9733	1962.933	1Oxidation 1Phospho	(F)SDVQFKMSHKRIMLF(T)
1962.9733	1962.933	1Oxidation 1Phospho	(N)LFSDVQFKMSHKRIM(L)

B

Ku70 S155A IR sample

Experimental (m/z)	Theoretical (m/z)	S/T modification	Sequence
513.31018	513.2854		(D)LMHL(K)
524.20166	524.1843		(F)QDMM(G)
537.31311	537.3031		(F)KNIY(V)
712.35645	712.3148		(F)YGTEKD(K)
723.36591	723.3494		(W)VC(Carbamidomethyl)ANLF(S)
855.44745	855.4247		(N)LFSDVQF(K)
1025.6068	1025.5514		(-)YISKIISSD(R)
1046.5553	1046.5598	1Oxidation	(F)KMSHKRIM(L)
1076.5413	1076.47	1Phospho	(D)YSLSEVLW(V)
1076.5413	1076.596		(F)KGQQGQKRF(Q)
1175.6117	1175.6129	1Oxidation	(D)TGIFLDLMHL(K)
1338.7183	1338.774		(E)LDNPGAKRILEL(D)
1353.6865	1353.7485		(L)QELDNPGAKRIL(E)
1401.6742	1401.6645		(F)YGTEKDKNSVNF(K)
1505.7886	1505.8079		(F)KMSHKRIMLFTN(E)
1522.7894	1522.8475		(-)YISKIISSDRDLL(A)
1580.8094	1580.705		(M)MGHGSDYSLSEVLW(V)
1602.8657	1602.8139	1Phospho	(-)YISKIISSDRDLL(A)
1769.9117	1769.8826	1Oxidation	(L)FSDVQFKMSHKRIM(L)
1783.8479	1783.9225		(L)LAVVFYGTEKDKNSVN(F)
1895.8875	1895.8309	1Oxidation	(D)VQFKMSHKRIMLFTN(E)
1895.8875	1895.9983	1Oxidation	(E)LDQFKGQQGQKRFQDM(M)
1969.9022	1969.9549		

Ku70 S155A NO IR sample

Experimental (m/z)	Theoretical (m/z)	S/T modification	Sequence
507.32974	507.3177		
524.20233	524.1843		(N)IYVL(Q)
550.34277	550.3235		(F)QDMM(G)
712.28613	712.3148		(D)TGIFL(D)
723.35962	723.3494		(F)YGTEKD(K)
745.33362	745.3168		(W)VC(Carbamidomethyl)ANLF(S)
1025.6021	1025.5514	1Phospho	(D)TGIFLD(L)
1175.6068	1175.6129		(-)YISKIISSD(R)
1338.7156	1338.774	1Oxidation	(D)TGIFLDLMHL(K)
1505.7819	1505.8079		(E)LDNPGAKRILEL(D)
1522.765	1522.8475		(F)KMSHKRIMLFTN(E)
1602.8645	1602.8139		(-)YISKIISSDRDLL(A)
1646.8513	1646.819	1Phospho	(-)YISKIISSDRDLL(A)
1769.9143	1769.8826	1Phospho	(N)SVNFKNIYVLQEL(D)
1783.8447	1783.9225	1Oxidation	(L)FSDVQFKMSHKRIM(L)
1969.9006	1969.9549		(L)LAVVFYGTEKDKNSVN(F)
2035.0427	2034.9233	1Oxidation	(E)LDQFKGQQGQKRFQDM(M)
504.289	504.2776		(F)KGQQGQKRFQDMMGHGSD(Y)
513.27612	513.2854		(L)QELD(N)
524.20337	524.1843		(D)LMHL(K)
529.28955	529.2803		(F)QDMM(G)
712.29852	712.3148	1Oxidation	(D)LMHL(K)
713.33014	713.2906		(F)YGTEKD(K)
723.36377	723.3494	1Phospho	(L)SEVLW(V)
738.34955	738.3305		(W)VC(Carbamidomethyl)ANLF(S)
745.31989	745.3168		(M)LFTNED(N)
1025.6003	1025.5514	1Phospho	(D)TGIFLD(L)
1175.6093	1175.6129		(-)YISKIISSD(R)
1338.7006	1338.774	1Oxidation	(D)TGIFLDLMHL(K)
1354.671	1354.7253		(E)LDNPGAKRILEL(D)
1401.6584	1401.6645		(D)LLAVVFYGTEKD(K)
1505.7809	1505.8079		(F)YGTEKDKNSVNF(K)
1580.8036	1580.705		(F)KMSHKRIMLFTN(E)
1602.86	1602.8139		(M)MGHGSDYSLSEVLW(V)
1613.8459	1613.7523	1Phospho	(-)YISKIISSDRDLL(A)
1613.8459	1613.8056	2Oxidation	(F)KGQQGQKRFQDMM(G)
1646.8459	1646.819	1Oxidation 1Phospho	(D)VQFKMSHKRIML(F)
1769.9038	1769.8826	1Phospho	(N)SVNFKNIYVLQEL(D)
1783.8328	1783.9225	1Oxidation	(L)FSDVQFKMSHKRIM(L)
2035.0067	2034.9233		(L)LAVVFYGTEKDKNSVN(F)

C

Ku70 162A IR sample

Experimental (m/z)	Theoretical (m/z)	S/T modification	Sequence
504.26657	504.2776		(E)KDKN(S)
513.25793	513.2854		(D)LMHL(K)
524.17059	524.1843		(F)QDMM(G)
529.26031	529.2803	1Oxidation	(D)LMHL(K)
623.32245	623.3035		(M)LFTNE(D)
668.33911	668.3436	1Oxidation	(D)VQFKM(S)
711.4118	711.4076		(L)LAVVFY(G)
723.3623	723.3494		(W)VC(Carbamidomethyl)ANLF(S)
738.32159	738.3305		(M)LFTNED(N)
745.35406	745.3168	1Phospho	(D)TGIFLD(L)
855.46509	855.4247		(N)LFSDVQF(K)
1014.5145	1014.502	1Phospho	(D)LRDTGIFL(D)
1025.6133	1025.5514		(-)YISKIISSD(R)
1064.5375	1064.4812	1Phospho	(N)SVNFKNIY(V)
1144.5438	1144.4654		(L)FTNEDNPHGN(D)
1175.6173	1175.6129	1Oxidation	(D)TGIFLDLMHL(K)
1338.7003	1338.774		(E)LDNPGAKRILEL(D)
1353.7028	1353.7485		(L)QELDNPGAKRIL(E)
1401.6826	1401.6645		(F)YGTEKDKNSVNF(K)
1406.6698	1406.6328	2Phospho	(Y)ISKIISSDRDL(L)
1505.7958	1505.8079	2Oxidation 2Phospho	(D)MMGHGSDYSLSE(V)
1522.7721	1522.8475		(-)YISKIISSDRDLL(A)
1580.8107	1580.705		(M)MGHGSDYSLSEVLW(V)
1597.8069	1597.7574	1Oxidation	(F)KGQQGQKRFQDMM(G)
1602.8734	1602.8139	1Phospho	(-)YISKIISSDRDLL(A)
1646.8423	1646.819	1Phospho	(N)SVNFKNIYVLQEL(D)
1769.9078	1769.8826	1Oxidation	(L)FSDVQFKMSHKRIM(L)
1783.8558	1783.9225		(L)LAVVFYGTEKDKNSVN(F)
1833.9534	1833.854	1Phospho	(L)FSDVQFKMSHKRIM(L)
1895.8716	1895.9983	1Oxidation	(D)VQFKMSHKRIMLFTN(E)
1962.9751	1962.933	1Oxidation 1Phospho	(L)FSDVQFKMSHKRIML(F)
1962.9751	1962.933	1Oxidation 1Phospho	(F)SDVQFKMSHKRIMLF(T)
1962.9751	1962.933	1Oxidation 1Phospho	(N)LFSDVQFKMSHKRIM(L)
2035.0055	2034.9233		(F)KGQQGQKRFQDMMGHGSD(Y)

Ku70 162A No IR sample

Experimental (m/z)	Theoretical (m/z)	S/T modification	Sequence
524.17334	524.1843		(F)QDMM(G)
537.31348	537.3031		(F)KNIY(V)
723.36835	723.3494		(W)VC(Carbamidomethyl)ANLF(S)
738.32764	738.3305		(M)LFTNED(N)
792.26416	792.2811	1Phospho	(F)YGTEKD(K)
1025.6025	1025.5514		(-)YISKIISSD(R)
1077.531	1077.5034		(L)SEVLWVC(Carbamidomethyl)AN(L)
1402.642	1402.6735	2Oxidation 1Phospho	(F)KMSHKRIMLF(T)
1466.7736	1466.7169	1Oxidation	(F)KGQQGQKRFQDM(M)
1466.7736	1466.7499		(L)DQFKGQQGQKRF(Q)
1505.7833	1505.8079		(F)KMSHKRIMLFTN(E)
1518.8159	1518.7716	1Phospho	(D)KNSVNFKNIYVL(Q)
1522.759	1522.8475		(-)YISKIISSDRDLL(A)
1580.8105	1580.705		(M)MGHGSDYSLSEVLW(V)
1597.8372	1597.7574	1Oxidation	(F)KGQQGQKRFQDMM(G)
1602.8577	1602.8139	1Phospho	(-)YISKIISSDRDLL(A)
1749.8984	1749.8775		(F)KMSHKRIMLFTNED(N)

	Gene	Fold S155D / WT expression Microarray 1	Microarray 2
**Cell cycle**	CDK1	-1.13272	-1.5631
	CDK11B	1.1717	-1.35505
	CDK12	-1.23528	-1.54936
	CDK14	-1.52117	-1.39001
	CDK17	-1.23738	-1.65717
	CDK2	-1.10442	-2.29363
	CDK20	1.30282	-1.34152
	CDK4	-1.21157	-1.62155
	CDK6	-1.71747	-1.62557
	CDK7	-1.12377	-1.94373
	CDK8	-1.04368	-1.64662
	CDK9	-1.06108	-1.41405
	Cdkn1a	1.78469	1.8669
	Cyclin B1	-1.02889	-1.73742
	Cyclin D1	/	-1.85051
	Cyclin D3	-1.08789	-1.19947
	Cyclin E1	/	-1.66409
	Cyclin F	-1.29918	-1.74507
	Cyclin J	-1.5685	-1.54095
	Cyclin Y	-1.5685	-2.47138
	Id1	-1.71293	-3.30425
	Id2	-1.54394	-2.97433
	Id3	-1.55826	-3.07059
	Inca1	2.08792	1.09476
**Apoptosis**	Atf3	2.29076	1.11201
	Ddit3	3.86476	2.64323
	Trp53inp1	3.92893	2.38246
	Xaf1	18.0812	9.14192
**Phosphatases**	Dusp6	-1.78878	-2.23834
	Ppp1ca	-1.04564	-1.51597
	Ppp1cb	-1.7477	-3.1441
	Ppp1cc	-1.03787	-1.31621
	Ppp2ca	-1.28673	-2.18602
	Ppp4r1	-1.27836	-1.30361
	Ppp4r4	-1.45548	-1.07197
	Ppp6r3	-1.27171	-1.20221
**Other**	Gadd45a	1.82471	4.08577
	Gadd45g	-1.30705	-1.70421
	Parp9	2.63466	1.53339
	Parp14	3.6658	3.17908
	Parp10	4.87628	4.04058

Supplementary Figure 2. S155D Ku70 induces expression changes in genes regulating cell cycle and apoptosis. Samples from WT and S155D Ku70 expressing MEFs were subjected to microarray analysis using a GeneChip Mouse Gene 1.0 ST Array (Affymetrics, Santa Clara, CA). GeneChips were processed at the London Regional Genomics Centre (Robarts Research Institute, London, ON; http://www.lrgc.ca). Shown is the list of genes, organized by cellular process, identified as differentially expressed in S155D Ku70 MEFs compared to the WT Ku70 control, with the fold expression change indicated for two separate experiments.

Supplementary Figure 3. Analysis of Ku70 S155D in DNA repair. (**A**) Ku70 S155D substitution does not induce DNA damage nor affects DNA repair efficiency. Pulsed-field gel electrophoresis (PFGE) analysis was performed on genomic DNA from Ku70$^{-/-}$ MEFs expressing wild-type Ku70 (WT), Ku70 S155D or empty pMSCV either untreated (control) or immediately after IR treatment (0 h) or 6 h following IR. For all samples, FAR (fraction of activity released) was averaged from three independent experiments, with error bars indicating SEM (*$p<0.05$). (**B**) DNA break assessment using alkaline (left) and neutral (right) comet assays. Untreated Ku70 WT and Ku70 S155D expressing MEFs, Ku70 WT expressing MEFs irradiated with 1 to 10 or 40 Gy respectively, and Ku70 WT MEFs treated with 100 μM hydrogen peroxide for 30 minutes were diluted and suspended in agarose gel on slides. Following lysis under alkaline or neutral conditions the slides were subjected to electrophoresis and stained with CYGREEN. Representative images (top) show DNA damage and corresponding migration. Scale bars, 10 mM. Mean tail moment was quantified for at least 100 comets using OpenComet, and mean tail moment was normalized to Ku70 WT expressing MEFs for each replicate. Normalized tail moment is shown with error bars indicating SD (n=3).

A

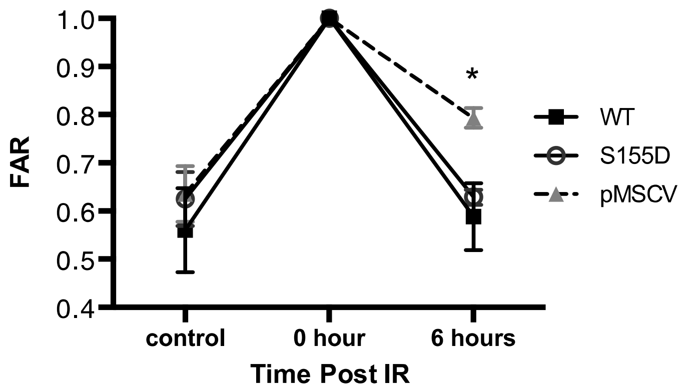

1.0
0.9
0.8
0.7
0.6
0.5
0.4
FAR
*
WT
S155D
pMSCV
control
0 hour
6 hours
Time Post IR

B

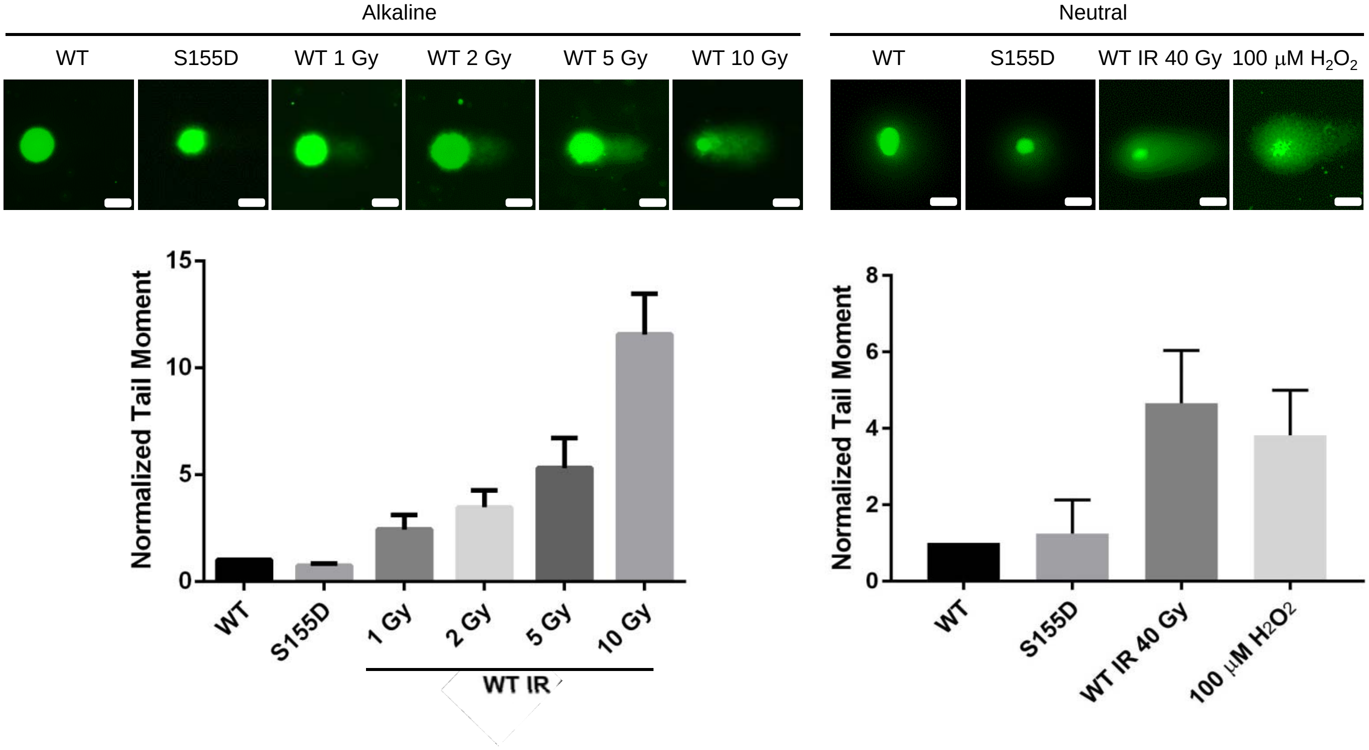

Alkaline
WT
S155D
WT 1 Gy
WT 2 Gy
WT 5 Gy
WT 10 Gy
Neutral
WT
S155D
WT IR 40 Gy
100 μM H_2O_2
Normalized Tail Moment
15
10
5
0
WT
S155D
1 Gy
2 Gy
5 Gy
10 Gy
WT IR
Normalized Tail Moment
8
6
4
2
0
WT
S155D
WT IR 40 Gy
100 μM H_2O_2

Supplementary Figure 4. (**A**) Silver stained gel of streptavidin-biotin pulldown of proteins interacting with Ku70 peptide containing either an alanine (A) or aspartic acid (D) substitution at the S155 position. Pull down was performed in MEF extracts that were either untreated or treated with 10 Gy of IR and incubated for 30 minutes. Band boxed in red was identified by MS/MS as being Aurora B. (**B**) Proteins were subjected to trypsin digestion and peptide masses obtained from MS/MS analysis. MASCOT (Matrix Science) database results for boxed band with parameters indicated.

A

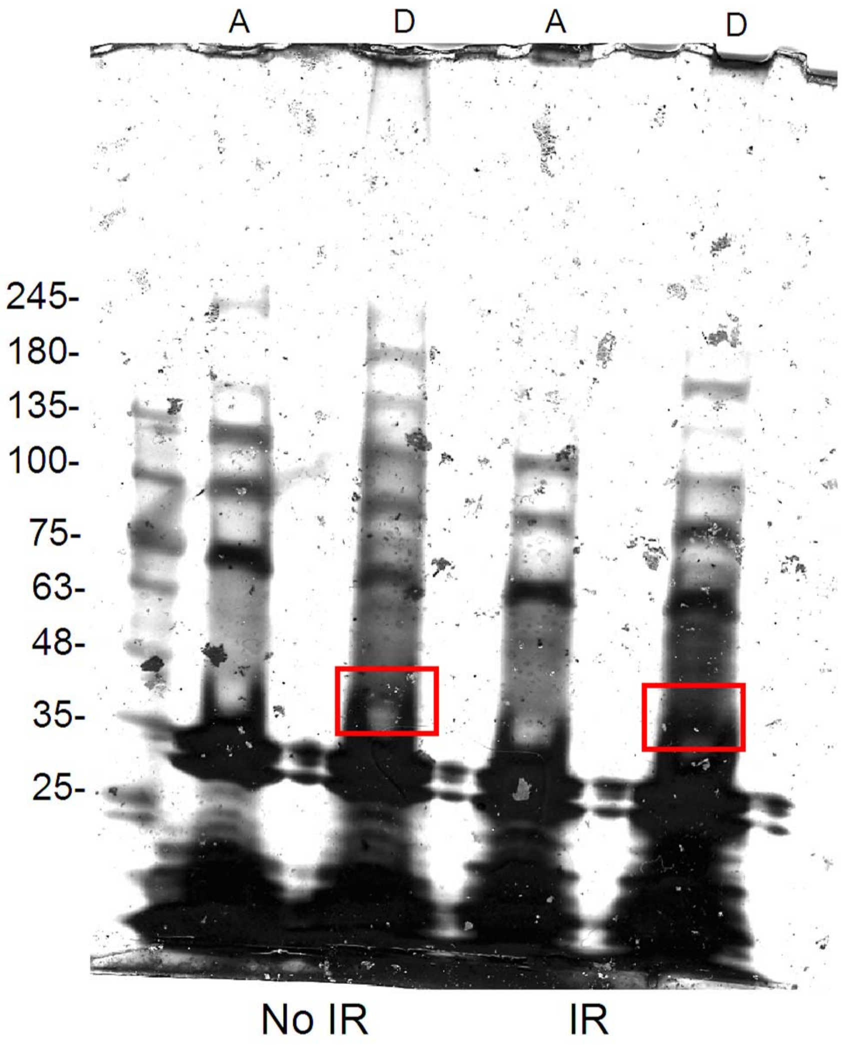

B

MASCOT Search Results

Protein View: gi|51317394

aurora kinase B [Mus musculus]

**Database:**	NCBInr
**Score:**	24
**Expect:**	5.1e+02
**Nominal mass (M_r):**	39588
**Calculated pI:**	9.47
**Taxonomy:**	Mus musculus

This protein sequence matches the following other entries:

- gi|341940262 from Mus musculus
- gi|26344858 from Mus musculus
- gi|74204872 from Mus musculus
- gi|148678525 from Mus musculus

Sequence similarity is available as an NCBI BLAST search of gi|51317394 against nr.

Search parameters

**Enzyme:**	
**Fixed modifications:**	Carbamidomethyl (C)
**Variable modifications:**	Oxidation (M)
**Mass values searched:**	76
**Mass values matched:**	4

Protein sequence coverage: 11%

Matched peptides shown in ***bold red***.

**1**	MAQKENAYPW	PYGSKTSQSG	LNTLSQRVLR	KEPATTSALA	LVNR**FNSQST**
**51**	**AAPGQKLAEN**	**K**SQGSTASQG	SQNKQPFTID	NFEIGRPLGK	GK**FGNVYLAR**
**101**	**EK**KSRFIVAL	KILFKSQIEK	EGVEHQLR**RE**	**IEIQAHLK**HP	NILQLYNYFY
**151**	DQQRIYLILE	YAPRGELYKE	LQKSRTFDEQ	RTATIMEELS	DALTYCHKKK
**201**	VIHRDIKPEN	LLLGLQGELK	IADFGWSVHA	PSLRRKTMCG	TLDYLPPEM
**251**	EGRMHNEMVD	LWCIGVLCYE	LMVGNPPFES	PSHSETYR**RI**	**VK**VDLKFPSS
**301**	VPSGAQDLIS	KLLKHNPWQR	LPLAEVAAHP	WVRANSRRVL	PPSAL

Unformatted sequence string: 345 residues (for pasting into other applications).

Sort peptides by ◉ Residue Number ○ Increasing Mass ○ Decreasing Mass

Show predicted peptides also

Start – End	Observed	Mr(expt)	Mr(calc)	ppm	M	Peptide
45 – 61	1790.8965	1789.8892	1789.8958	-3.70	1	R.FNSQSTAAPGQKLAENK.S
93 – 102	1196.6475	1195.6402	1195.6349	4.43	1	K.FGNVYLAREK.K
129 – 138	1236.7520	1235.7447	1235.6986	37.3	1	R.REIEIQAHLK.H
289 – 292	515.3500	514.3427	514.3591	-31.9	1	R.RIVK.V

No match to: 522.1439, 524.1525, 526.1677, 527.3412, 532.1259, 544.2593, 546.1281, 550.1316, 552.1475, 562.2569, 568.1403, 570.1439, 571.3336, 574.6748, 590.1094, 590.2841, 591.3544, 592.0972, 603.2925, 606.3408, 612.0964, 614.1000, 617.0551, 618.0634, 621.3695, 634.0793, 636.0825, 637.3045, 644.3737, 650.0524, 652.0555, 659.2951, 691.0883, 693.0884, 705.3827, 780.4994, 801.1160, 823.1085, 825.1118, 830.4670, 835.5049, 839.0843, 841.0833, 842.5198, 845.1009, 847.0994, 854.5222, 856.5255, 861.0728, 867.4332, 870.5452, 943.5807, 945.5692, 956.3621, 959.0843, 969.5425, 976.4651, 1027.5116, 1034.1311, 1036.1300, 1045.5720, 1050.1051, 1052.1104, 1057.5663, 1132.5400, 1154.5925, 1155.6062, 1176.5413, 1179.6077, 1252.6168, 1493.7444, 1726.7675

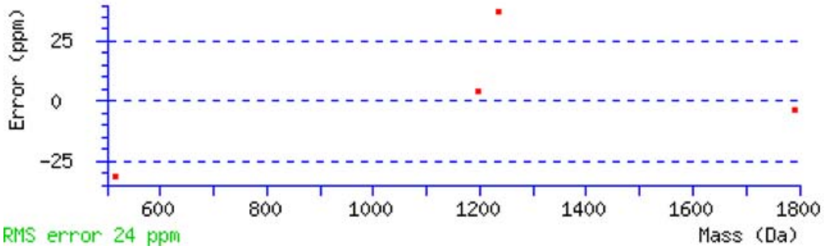

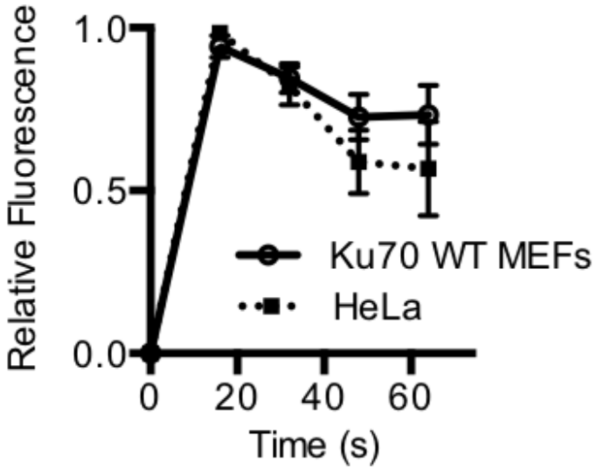

Supplementary Figure 5. Quantification of Aurora B accumulation at sites of laser microirradiation. GFP-Aurora B was transfected in Ku70 WT MEFs and in Hela cells and GFP-Aurora B-expressing cells were subjected to laser microirradiation. Shown are quantifications of Aurora B recruitment to the path of laser damage in MEFs (n=10) and Hela cells (n=5). Plotted is the relative fluorescence of GFP over time after laser exposure. Error bars indicate SEM

Supplementary Materials and Methods

Mass Spectrometry and Protein Identification.

For phosphorylation identification, 70% confluent MEFs were mock-treated or treated with 40 Gy of IR and incubated for 30 minutes. 10 mg of nuclear extracts were adjusted to 100 mM KCl, 2% NP-40 and immunoprecipitated as described above with a Ku70 antibody (N3H10, Santa Cruz). Immunoprecipitates were boiled in 1X SDS loading buffer (2% SDS, 2mM DTT, 5% Glycerol, 40 mM Tris-HCl, 0.01% bromophenol blue), run on an SDS-PAGE gel and stained with Coomasssie Blue G-250 (Protea Biosciences, Morgantown, WV).

For peptide pull-down and protein identification, 4 mg of untreated or 10 Gy-treated MEF extracts were incubated for 2 hours with one of the following N-terminal biotin-conjugated peptides (Genscript, Piscataway, NJ): biotin-EVLWVCANLFADVQFKMSH, biotin-EVLWVCANLFDDVQFKMSH. 10 μg of peptide was pre-coupled to 40 μl of Steptavidin beads (Invitrogen) for 30 min at room temperature. Pre-coupled beads and extracts were incubated for 2 hours at 4°C. Beads were washed in (Tris buffer saline, 0.1% Tween20), boiled in 1X SDS loading buffer and run on SDS-PAGE gel. Gels were stained with the Silver Stain Plus Kit (Bio-Rad) as per manufacturer instruction.

In-gel digestion was performed using a MassPREP automated digester station (PerkinElmer, Waltham, MA). Gel pieces were Coomassie de-stained using 50 mM ammonium bicarbonate and 50% acetonitrile or silver de-stained using a 50 mM sodium thiosulphate 5 hydrate and 15 mM potassium ferricyanide solution, which was followed by protein reduction using 10 mM dithiotreitol (DTT), alkylation using 55 mM iodoacetamide (IAA), and tryptic or chymotryptic digestion. Peptides were extracted using a solution of 1% formic acid and 2% acetonitrile and

lyophilized. Prior to mass spectrometric analysis, dried peptide samples were re-dissolved in a 10% acetonitrile and 0.1% TFA (trifluoroacetic acid) solution.

Mass Spectrometry data were obtained using an AB Sciex 5800 TOF/TOF System, MALDI TOF TOF (Framingham, MA, USA). Data acquisition and data processing were respectively done using a TOF TOF Series Explorer and Data Explorer (AB Sciex). The instrument is equipped with a 349 nm OptiBeam On-Axis laser. The laser pulse rate is 400 Hz. Reflectron positive mode was used. Reflectron mode was externally calibrated at 50 ppm mass tolerance and internally at 10 ppm. Each mass spectrum was collected as a sum of 400 shots. MALDI matrix, α-cyano-4-hydroxycinnamic acid (CHCA), was prepared as 5 mg/mL in 6mM ammonium phosphate monobasic, 50% acetonitrile, 0.1 % trifluoroacetic acid and mixed with the sample at 1:1 ratio (v/v). All procedures were performed at the London Regional Proteomics Centre (LRPC).

Pulsed-field Gel Electrophoresis (PFGE). One day prior to irradiation, 3.5 million cells were seeded onto a 10 cm plate. Following 40 Gy of IR or mock treatment, cells were harvested into agarose plugs using the Bio-Rad CHEF Genomic DNA Plug Kit. Agarose plugs were run on a 0.8% gel using the Bio-Rad CHEF-DR II system (200-500s switch time, 120° angle, 3V/cm, 48 hours). The gels were stained with Red Safe (Frogga Bio), images captured using a BioRad ChemiDoc and ImageLab software and staining was quantified using ImageJ. The fraction released (fraction of activity released, FAR) corresponding to unrepaired DNA was calculated by calculating the ratio of the DNA migrating below the plug over the total DNA loaded (DNA remaining in the plug and fraction entering the gel).

Alkaline and Neutral Comet Assay. For both assays, Ku70 WT expressing MEFs either untreated, treated with 100 μM hydrogen peroxide, irradiated with 1, 2, 5 and 10 Gy for alkaline assay or irradiated with 40 Gy for neutral assay and Ku70 S155D expressing MEFs were collected by scraping and were suspended in low melting point agarose on glass slides. The neutral comet assay was done using the Enzo Comet SCGE assay kit (Enzo Life Sciences) following the product manual. Briefly, the cells embedded in agarose were incubated in lysis solution for 30 minutes followed by electrophoresis. Slides were dried and stained with CYGREEN. Alkaline comet assays were performed by lysing gel embedded cells for 1h followed by a 45 min incubation in pH 13 buffer and brief electrophoresis (in the dark). Slides were neutralized and stained with CYGREEN followed immediately by visualization. Pictures were taken with an Olympus BX51 microscope at 40x magnification using the Image-Pro Plus software (Media Cybernetics, Inc., Bethesda, MD). Comets were scored using OpenComet, a plugin for ImageJ software. Mean tail length was normalized to Ku70 WT expressing MEFs for each replicate.

Primers for RT-PCR:

Cyclin B1:	Forward	'ATCGGGGAACCTCTGATTTT'
	Reverse	'TCACACACAGGCACCTTCTC'
Cyclin D1:	Forward	'GCGTACCCTGACACCAATCTC'
	Reverse	'CTCCTCTTCGCACTTCTGCTC'
PP1c:	Forward	'GATGTCGTCCAGGAAAGATTGT'
	Reverse	'TCAGTGGTGCTTCCAATTCCA'
Xaf1:	Forward	'AGCCATGTGTCTGAGTGCAAA'
	Reverse	'GCAAAGATCACAACGGGTTTTTC'

CDK6:	Forward 'GGCGTACCCACAGAAACCATA'
	Reverse 'AGGTAAGGGCCATCTGAAAACT'
